# Cross-seeding of prions by aggregated α-synuclein leads to transmissible spongiform encephalopathy

**DOI:** 10.1371/journal.ppat.1006563

**Published:** 2017-08-10

**Authors:** Elizaveta Katorcha, Natallia Makarava, Young Jin Lee, Iris Lindberg, Mervyn J. Monteiro, Gabor G. Kovacs, Ilia V. Baskakov

**Affiliations:** 1 Center for Biomedical Engineering and Technology, University of Maryland School of Medicine, Baltimore, Maryland, United States of America; 2 Department of Anatomy and Neurobiology, University of Maryland School of Medicine, Baltimore, Maryland, United States of America; 3 Institute of Neurology, Medical University of Vienna, Vienna, Austria; University of Alberta, CANADA

## Abstract

Aggregation of misfolded proteins or peptides is a common feature of neurodegenerative diseases including Alzheimer’s, Parkinson’s, Huntington’s, prion and other diseases. Recent years have witnessed a growing number of reports of overlap in neuropathological features that were once thought to be unique to only one neurodegenerative disorder. However, the origin for the overlap remains unclear. One possibility is that diseases with mixed brain pathologies might arise from cross-seeding of one amyloidogenic protein by aggregated states of unrelated proteins. In the current study we examined whether prion replication can be induced by cross-seeding by α-synuclein or Aβ peptide. We found that α-synuclein aggregates formed in cultured cells or *in vitro* display cross-seeding activity and trigger misfolding of the prion protein (PrP^C^) in serial Protein Misfolding Cyclic Amplification reactions, producing self-replicating PrP states characterized by a short C-terminal proteinase K (PK)-resistant region referred to as PrPres. Non-fibrillar α-synuclein or fibrillar Aβ failed to cross-seed misfolding of PrP^C^. Remarkably, PrPres triggered by aggregated α-synuclein *in vitro* propagated in animals and, upon serial transmission, produced PrP^Sc^ and clinical prion disease characterized by spongiosis and astrocytic gliosis. The current study demonstrates that aggregated α-synuclein is potent in cross-seeding of prion protein misfolding and aggregation *in vitro*, producing self-replicating states that can lead to transmissible prion diseases upon serial passaging in wild type animals. In summary, the current work documents direct cross-seeding between unrelated amyloidogenic proteins associated with different neurodegenerative diseases. This study suggests that early interaction between unrelated amyloidogenic proteins might underlie the etiology of mixed neurodegenerative proteinopathies.

## Author summary

Aggregation of misfolded proteins or peptides is a common feature of neurodegenerative diseases. Recent years have witnessed a growing number of reports of overlap in neuropathological features specific to two or more neurodegenerative diseases in individual patients. However, the origin for the overlap remains unclear. One possibility is that disease that have mixed brain pathologies might arise from cross-seeding of one amyloidogenic protein by fibrillar states of unrelated proteins. The current study examined whether prion replication can be induced by cross-seeding by α-synuclein or Aβ peptide in their aggregated states. We found that α-synuclein aggregates display cross-seeding activity and trigger misfolding of the prion protein (PrP^C^) *in vitro*, producing self-replicating PrP states. Non-fibrillar α-synuclein or fibrillar Aβ failed to cross-seed misfolding of PrP^C^. Remarkably, misfolded PrP triggered by fibrillar α-synuclein *in vitro* propagated in animals and, upon serial transmission, produced clinical prion diseases. In summary, the current work documents direct cross-seeding between unrelated amyloidogenic proteins associated with different neurodegenerative diseases. This study suggests that early interaction between unrelated amyloidogenic proteins might underlie the etiology of mixed neurodegenerative proteinopathies.

## Introduction

Aggregation of misfolded proteins or peptides is a common feature of neurodegenerative diseases including Alzheimer’s, Parkinson’s, Huntington’s, prion and other diseases [1,2]. According to traditional view, each neurodegenerative disease is characterized by aggregation of one or two disease-specific proteins or peptides, for instance, Aβ and tau in Alzheimer’s disease, α-synuclein in Parkinson’s disease, or prion protein in prion diseases. In recent years, however, an increasing number of studies have revealed that some individuals show co-occurrence of neuropathological features characteristic of more than one neurodegenerative disease [3–8] (reviewed in [9]). For example, α-synuclein pathology (Lewy bodies) can be detected in Creutzfeldt-Jakob disease (CJD) patients including sporadic and genetic forms [7,10,11]. Moreover, histological examination demonstrated that aggregates of unrelated amyloidogenic proteins or peptides, including prion protein, tau, Aβ peptides, α-synuclein, immunoglobulin light chain λ, and β_2_-microglobulin, could be observed within the same plaques or in close proximity [12–17]. However, the mechanisms responsible for co-aggregation of different amyloidogenic proteins are not understood. Could cross-seeding between unrelated amyloidogenic proteins contribute to the etiology of neurodegenerative diseases?

Two different mechanisms have been proposed to explain how diseases with mixed brain pathologies or symptoms may arise. According to one mechanism, a general decline in proteostasis including ER stress, impairment of proteasome-, lysosome-, or autophagosome-dependent degradation during normal ageing or disease conditions, could trigger co-aggregation of multiple amyloidogenic proteins that are prone to misfolding. Such a mechanism assumes that imposing a stress on proteostasis results in misfolding and aggregation of multiple proteins independently or semi-independently, i.e. in the absence of direct cross-seeding. Consistent with this hypothesis is a study in which shorter incubation times to prion disease were observed in transgenic mice that overexpress human A53T α-synuclein compared to non-transgenic controls inoculated with three prion strains [18]. Another study reported that inoculation of prions into aged transgenic mice overexpressing human wild type α-synuclein resulted in more extensive and abundant intraneuronal and synaptic accumulation of α-synuclein relative to non-transgenic control mice [19]. In addition, in agreement with the latter mechanism is the observation that CJD patients exhibit impairments of the nigrostriatal pathway, which is a hallmark of Parkinson’s disease [7].

An alternative mechanism proposes that co-occurrence of mixed brain pathologies arises by direct cross-seeding of protein aggregates of one disease-related protein by fibrils or oligomers of an unrelated protein. A few studies have provided experimental evidence in support of this idea. Examples of cross-talk between different yeast prion proteins in a cell were documented more than a decade ago [20,21]. In addition, reactive protein A amyloidosis or senile apolipoprotein A-II amyloidosis were found to develop in mice as a result of cross-seeding by fibrils of apolipoprotein A-II or protein A, respectively [22]. In recent studies, examples of cross-seeding of Tau by Aβ fibrils or α-synuclein fibrils were illustrated using *in vitro*, cellular and animal models [23–25].

In this study we examined whether prion replication can be induced by cross-seeding with aggregated α-synuclein or Aβ peptide. We found that α-synuclein aggregates formed either in cultured cells or *in vitro* triggered misfolding of PrP^C^ in serial Protein Misfolding Cyclic Amplification reactions, producing self-replicating PrP states characterized by a short C-terminal proteinase K (PK) resistant region referred to as PrPres. Non-fibrillar α-synuclein or fibrillar Aβ failed to cross-seed misfolding of PrP^C^. Remarkably, PrPres triggered by aggregated α-synuclein *in vitro* propagated in animals and, upon serial transmission, produced PrP^Sc^ and clinical prion disease characterized by spongiosis and astrocytic gliosis. The current study demonstrates that aggregated α-synuclein is potent in cross-seeding misfolding and aggregation of the prion protein *in vitro*, producing self-replicating states that can lead to transmissible prion diseases upon serial passage in wild type animals. In summary, this work documents direct cross-seeding between unrelated amyloidogenic proteins associated with different neurodegenerative diseases.

## Results

**Fig 1:**
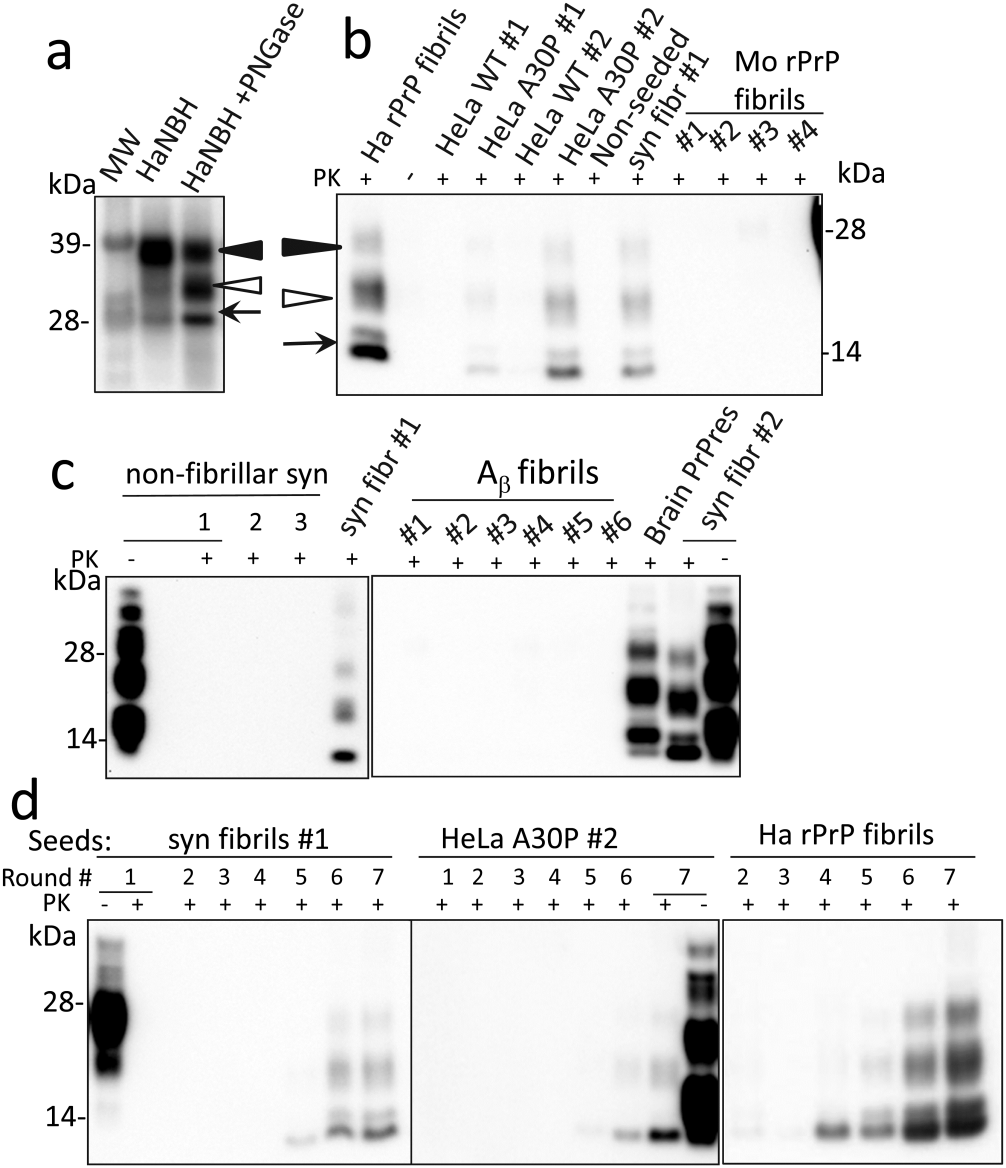
Cross-seeding of PrP^C^ misfolding and replication in dgPMCAb. **a** Analysis of the glycoform ratios of PrP^C^ from Syrian hamster normal brain homogenate (HaNBH) before and after treatment with PNGase F. Black and white triangles mark di- and monoglycosylated glycoforms, respectively, whereas arrows mark the unglycosylated form. **b** and **c** Serial dgPMCAb reactions were seeded as labeled with (i) lysates of HeLa cells expressing WT α-synuclein (HeLa WT) or A30P mutant (HeLa A30P) and cultured under two conditions (#1, #2) as described in Methods; (ii) amyloid fibrils produced *in vitro* from recombinant α-synuclein from two sources (syn fibrils #1, #2 as described in Methods); (iii) mouse rPrP amyloid fibrils produced *in vitro* in 0, 0.1, 0.5, or 2.5M GdnHCl (Mo rPrP fibrils #1, #2, #3, #4, respectively); (iv) non-fibrillar α-synuclein (non-fibr); or (v) Aβ fibrils produced *in vitro* using six different protocols as described in Methods (Aβ fibrils, #1–#6). As a control for cross-contamination, non-seeded dgPMCAb reactions were conducted in parallel (non-seeded). Seven serial dgPMCAb rounds with 10-fold dilutions between rounds were conducted and the products of the seventh round were treated with PK and analyzed by Western blots using SAF-84 antibodies. As references, dgPMCAb-derived PrPres formed in serial dgPMCAb seeded with hamster rPrP fibrils produced *in vitro* in 0.5 M GdnHCl (Ha rPrP fibrils, panel **b**) or brain- derived PrPres from animals inoculated with hamster rPrP fibrils are provided (brain PrPres, panel **c**) [26]. **d** Analysis of PrPres dynamics in serial dgPMCAb reactions seeded with α-synuclein WT fibrils #1, lysates of HeLa cells expressing α-synuclein A30P variant and cultured under condition #2, or hamster rPrP fibrils.

To examine the ability of α-synuclein to cross-seed aggregation of PrP^C^, a Protein Misfolding Cyclic Amplification with beads that employs partially deglycosylated Syrian hamster PrP^C^ as a substrate (dgPMCAb) was used [26,27]. Partial deglycosylation by treatment with PNGase F altered the ratio of the three PrP^C^ glycoforms in favor of mono- and unglycosylated PrP^C^ at the expense of diglycosylated PrP^C^ (Fig 1a). Previously we showed that partial deglycosylation of PrP^C^ removes spatial constraints imposed by bulky N-linked carbohydrates, expanding the range of possible folding patterns that PrP^C^ can acquire upon conversion into self-replicating, PrP^Sc^-like states [26–28]. As an illustration of this effect, amyloid fibrils prepared from recombinant hamster PrP *in vitro* failed to seed prion replication in Protein Misfolding Cyclic Amplification with beads (PMCAb) that used non-treated PrP^C^, but displayed consistent seeding activity in dgPMCAb with partially deglycosylated PrP^C^ [26,28].

To examine cross-seeding activity, lysates of HeLa cells transfected with either GFP-tagged wild type (WT) α-synuclein or with the Parkinson’s disease (PD) A30P mutant α-synuclein were assessed for their ability to seed PrP^C^ conversion in serial dgPMCAb reactions consisted of seven serial rounds. Preliminary studies revealed that, in contrast to WT α-synuclein, the A30P mutant formed aggregates in HeLa cells (S2a and S2b Fig). Remarkably, misfolded PK-resistant PrP states were formed in serial dgPMCAb reactions seeded with lysates of cells expressing the A30P mutant, but not WT α-synuclein (Fig 1b). The dgPMCAb-derived PK-resistant PrP products will be referred to as PrPres. The dgPMCAb-derived PrPres consisting of di-, mono-, and unglycosylated bands were detectable by the SAF-84 antibody, which recognizes the C-terminal epitope 160–170. Notably, the PK resistance pattern of dgPMCAb-derived PrPres was similar to PrPres formed in dgPMCAb reactions seeded with Syrian hamster (Ha) full-length recombinant PrP (rPrP) fibrils described in previous studies (Fig 1b) [26]. To test whether seeding activity is attributable to aggregated forms of α-synuclein, recombinant α-synuclein obtained from two independent sources described in Materials and Methods was converted *in vitro* into amyloid fibrils and tested in serial dgPMCAb (S1 Table, S2c Fig). Fibrillar preparations of α-synuclein from both sources consistently showed cross-seeding activity in serial dgPMCAb in three independent experiments, whereas all reactions seeded with non-fibrillar α-synuclein were negative (Fig 1b and 1c). All experiments were performed using equipment and laboratory space that have never been exposed to prions. Nevertheless, as negative controls, non-seeded dgPMCAb reactions were conducted in parallel in each experiment and were always negative (Fig 1b).

To examine the specificity of PrP^C^ cross-seeding by α-synuclein, serial dgPMCAb was seeded with Aβ amyloid fibrils prepared *in vitro* under six different solvent conditions using Aβ(1–40) peptide (S1 Table, S2d Fig). All dgPMCAb reactions seeded with Aβ fibrils were negative (Fig 1c). To further establish the specificity of cross-seeding, dgPMCAb reactions were seeded with amyloid fibrils prepared *in vitro* under four different solvent conditions using mouse (Mo) full-length rPrP rather than Ha rPrP (S1 Table). While the sequences of Mo and Ha full-length PrPs are 94% identical (S3 Fig), our previous studies established that the fibrils formed by two rPrP variants are structurally different [29,30]. Serial dgPMCAb reactions seeded with Mo rPrP fibrils were all negative (Fig 1b). In summary, in addition to Ha rPrP fibrils, only α-synuclein in aggregated states (produced in *vitro* or by cultured cells, respectively) showed cross-seeding activity in the serial dgPMCAb assay.

The relative efficiency of cross-seeding or the amounts of active seeds could be estimated from a time-point or a round number at which the first PrPres could be detected by Western blot. When seeded with Ha rPrP fibrils, the conversion products were detected by the third or fourth dgPMCAb rounds (Fig 1d and [26]). In the reactions seeded by fibrillary α-synuclein, the first PrPres was visible by the fifth round. As expected, this result illustrates that the cross-seeding of PrP^C^ aggregation by α-synuclein fibrils was less efficient than seeding by Ha rPrP fibrils.

**Fig 2:**
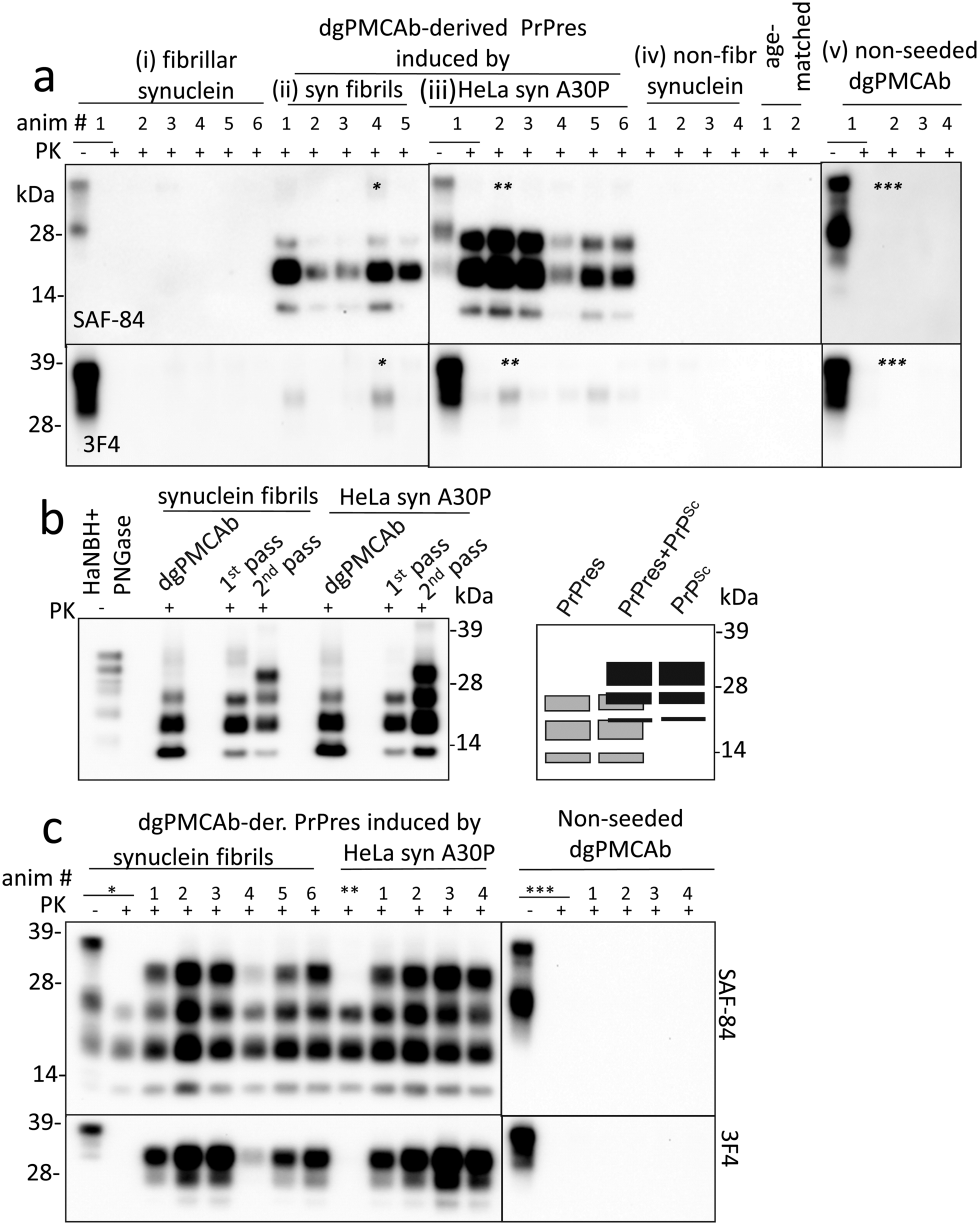
Bioassay of dgPMCAb-derived PrPres in Syrian hamsters. **a** Western blot analysis of brain material from hamsters inoculated IC with (i) fibrillar α-synuclein, (ii, iii) dgPMCAb-derived PrPres induced by WT α-synuclein fibrils or lysates of HeLa cells expressing the A30P variant, (iv) non-fibrillar α-synuclein, or (v) non-seeded dgPMCAb-derived material and stained with SAF-84 (top panels) or 3F4 antibody (bottom panels). Non-inoculated, age-matched animals were examined as a negative control. Brain materials marked by asterisks were used for the second passage. **b** Left panel shows Western blot analysis of dgPMCAb-derived PrPres used for inoculation and resulting brain-derived PrPres and PrP^Sc^ from animals of the 1^st^ and 2^nd^ passages. To match the amounts of dgPMCAb- and brain-derived PrPres on a blot, all brain materials were diluted 250-fold after PK treatment. Western blots were stained with SAF-84 antibody. On the right panel is a schematic representation of the PK resistant profile showing overlap between the three glycoforms of PrPres (gray boxes) and the three glycoforms of PrP^Sc^ (black boxes). **c** Western blot analysis of brain material from hamsters inoculated with the second passage of dgPMCAb-derived PrPres induced by α-synuclein fibrils; lysates of HeLa cells expressing the A30P mutant; or non-seeded dgPMCAb-derived material. Lanes marked by asterisks show brain material from animals from the first passages used for serial transmission. Western blots were stained with SAF-84 (top panels) or 3F4 antibody (bottom panels).

To test whether dgPMCAb-derived PrPres induced prion disease in animals, Syrian hamsters were inoculated with the products of serial dgPMCAb reactions seeded with (i) the lysates of HeLa cells expressing α-synuclein A30P or (ii) WT α-synuclein fibrils produced *in vitro* (Table 1). The products of non-seeded serial dgPMCAb reactions were inoculated as a negative control. In addition, to examine whether α-synuclein cross-seeded prion replication directly in animals, two animal groups were inoculated with either fibrillar or non-fibrillar WT α-synuclein (Table 1). No obvious clinical signs were detected in any animal groups, and all animals were euthanized at 561 days after inoculation (Table 1). Nevertheless, all animals inoculated with PrPres products from dgPMCAb reactions seeded with fibrillar WT α-synuclein fibrils or lysates of HeLa cells expressing α-synuclein A30P mutant showed PrPres (Fig 2a, Table 1). Remarkably, the PK-digestion pattern of the animal-derived PrPres was very similar, but not identical, to that of dgPMCAb-derived PrPres (Fig 2b). The animal-derived PrPres consisted of predominant monoglycosylated and smaller amounts of di- and unglycosylated bands that can be detected by C-terminal SAF-84 antibody (epitope 160–170) (Fig 2a). The animal-derived PrPres was not detectable by 3F4 antibody immunoreactive to the epitope 109–112 (Fig 2a). The total amount of brain-derived PrPres exceeded more than 10^3^-fold the amount of PrPres in the inocula arguing that PrPres was able to replicate effectively in animals. In addition to PrPres, very small amounts of PrP^Sc^ detectible by 3F4 were found in animals in both groups (Fig 2a). None of the animals inoculated with fibrillar or non-fibrillar WT α-synuclein or dgPMCAb-derived material from non-seeded reactions displayed any PK-resistant products, as judged by SAF-84 or 3F4 staining (Fig 2a, Table 1). In summary, PrPres generated *in vitro* under conditions with altered PrP^C^ glycoform ratios were able to propagate in animals despite unfavorable ratios of di-, mono- and unglycosylated PrP^C^ resulting in accumulation of animal-derived PrPres and small amounts of PrP^Sc^.

**Table 1:** Bioassay in Golden Syrian hamsters.

Material used for inoculation	PKres in dgPMCAb	1^st^ passage ^c^		2^nd^ passage ^d^		
		n_s_/n_t_^a^	n_PK-res_/n_t_^b^	n_s_/n_t_^a^	n_PK-res_/n_t_^b^	Clinical disease, dpi
dgPMCAb products seeded with A30P α-synuclein HeLa #2	yes	0/6	6/6	5/5	5/5	588±19
dgPMCAb products seeded with fibrillar α-synuclein (source #2)	yes	0/5	5/5	6/6	6/6	607±21
non-seeded dgPMCAb	no	0/4	0/4	0/4	0/4	none
dgPMCAb products seeded with non-fibrillar α-synuclein (source #2)	no	-	-	-	-	-
fibrillar α-synuclein (source #2)		0/6	0/6	-	-	-
non-fibrillar α-synuclein (source #2)		0/4	0/4	-	-	-

^a^ Number of animals with clinical signs over the total number of animals survived to the end of the experiment.

^b^ Number of animals with PrPres in brains by Western blot over the total number of animals which survived to the end of the experiment

^c^ The animals from the 1^st^ passage were euthanized at 561 days postinoculation

^d^ The animals from the 2^d^ passage were euthanized at 638–642 days postinoculation

**Fig 3:**
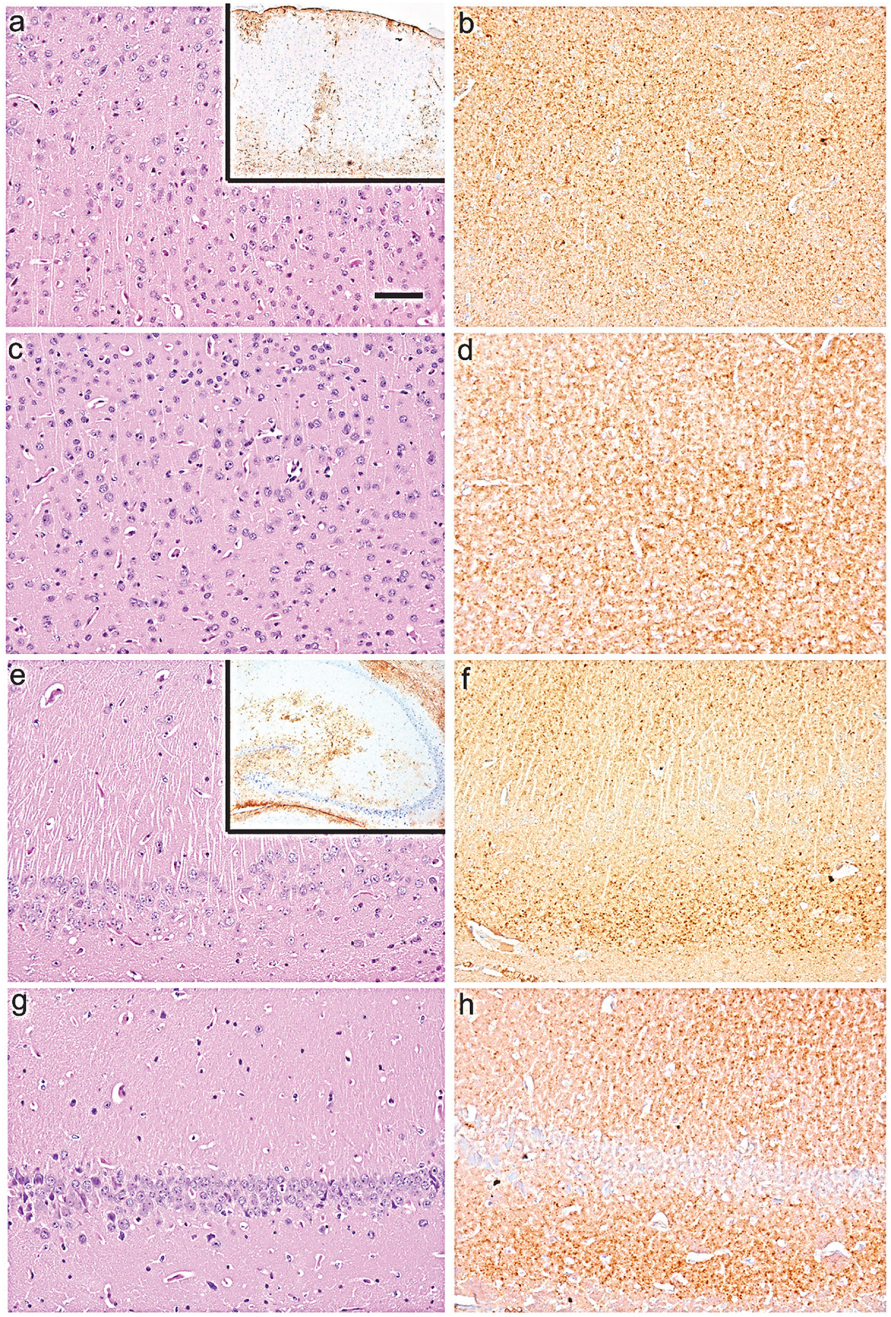
Histopathological analysis of brains from the 1^st^ passage of dgPMCAb-derived PrPres. Representative images of the frontal cortex (**a, b, c, d**) and hippocampus (**e, f, g, h**) of animals inoculated with dgPMCAb products seeded with fibrillar WT α-synuclein (**a,b,e,f**) or dgPMCAb products seeded with lysates of HeLa cells expressing A30P α-synuclein (**c,d,g,h**). Note the lack of spongiform change in the sections stained with hematoxylin and eosin (**a, c, e, g**) and patchy reactive astrogliosis (immunostaining for GFAP are in the insets in panels **a** and **c**). Immunostaining for PrP using SAF-84 (**b, d, f, h**) revealed diffuse/synaptic and granular deposits. Scale bar in **a** = 50 μm.

Histopathological evaluation of two animal groups inoculated with dgPMCAb-derived PrPres revealed mild focal reactive astrogliosis in all examined animals mostly in the hippocampus and variably in other regions such as frontal cortex or thalamus (Fig 3, S4 Fig). Typical spongiform changes associated with prion diseases were not evident, although single small vacuoles were found in certain regions of the brain. Immunostaining for PrP using SAF-84 revealed granular or fine diffuse synaptic deposits, but no plaques or amorphous deposits (Fig 3, S4 Fig). PrP immunoreactivity was seen predominantly in the cortex and hippocampus and variably in subcortical areas. Consistent with very minor amounts of 3F4-positive PrP^Sc^ detectable by Western blot in animals of these groups, immunostaining for PrP using 3F4 did not reveal unequivocal pathological deposits (S5 Fig). Overall, histopathological analysis showed similar changes in animals of both groups that involved fine diffuse/synaptic SAF-84-positive PrP immunoreactivity, minor astrocytic gliosis, minimal if any spongiosis and lack of microgliosis (Fig 3, S6 Fig). Control groups including animals inoculated with products of non-seeded dgPMCAb reactions, WT α-synuclein fibrils or non-fibrillar α-synuclein also showed only minor GFAP staining, no significant vacuolization, and lack of microgliosis as well as lack of PrP deposits, as probed by SAF-84 (S7 Fig). In summary, both animal groups inoculated with dgPMCAb-derived PrPres showed limited prion pathology as judged by Western blot and histopathological analysis, but no clinical disease.

To test whether animals inoculated with dgPMCAb-derived PrPres might develop α-synucleinopathy due to possible amplification of α-synuclein aggregates in dgPMCAb, the animal group injected with dgPMCAb-derived PrPres seeded with fibrillary WT α-synuclein and un-inoculated age-matched control group were also examined by staining with anti-α-synuclein antibodies. Immunostaining for α-synuclein with antibody 4D6 revealed prominent synaptic α-synuclein immunoreactivity in animals of both groups (S8 Fig), which is consistent with its normal physiologic distribution. Synaptic immunoreactivity was more prominent in areas with larger synaptic boutons (e.g. cerebellum granular layer or hippocampus) (S8a–S8f Fig). In addition, coarse α-synuclein deposits and very rare diffuse neuronal cytoplasmic staining were detected in all examined animals of both groups, however, no unequivocal Lewy-body like inclusions were found (S8 Fig). While we cannot exclude the possibility that these coarser deposits represent early aggregates, these findings should be interpreted with great caution, as it is currently unclear whether Syrian hamsters can develop any human-like α-synuclein pathology.

To examine whether serial transmission of brain material containing PrPres leads to clinical prion disease, brain material from animals inoculated with dgPMCAb-derived PrPres induced either by α-synuclein WT fibrils, the lysates of HeLa cells expressing α-synuclein A30P, or non-seeded dgPMCAb-derived material were used for the second passage (Table 1). The animal groups that were injected with brain material containing PrPres developed clinical symptoms including hyperreactivity, dry skin, rough patchy coat and dry eyes (Table 1). All animals from these two groups showed substantial amounts of PrPres detectible by SAF-84 and PrP^Sc^ that was detected by both SAF-84 and 3F4 antibody (Fig 2c). Assessment of the dynamics revealed that PrPres continued to propagate during serial transmission, whereas the amounts of PrP^Sc^ increased substantially in the course of the second passage relative to those found in the first passage (Fig 2b). The control group of animals, which is the second passage of non-seeded dgPMCAb-derived material, did not display any clinical signs (Table 1). Brain material from this group did not contain any PK-resistant products (Fig 2c).

**Fig 4:**
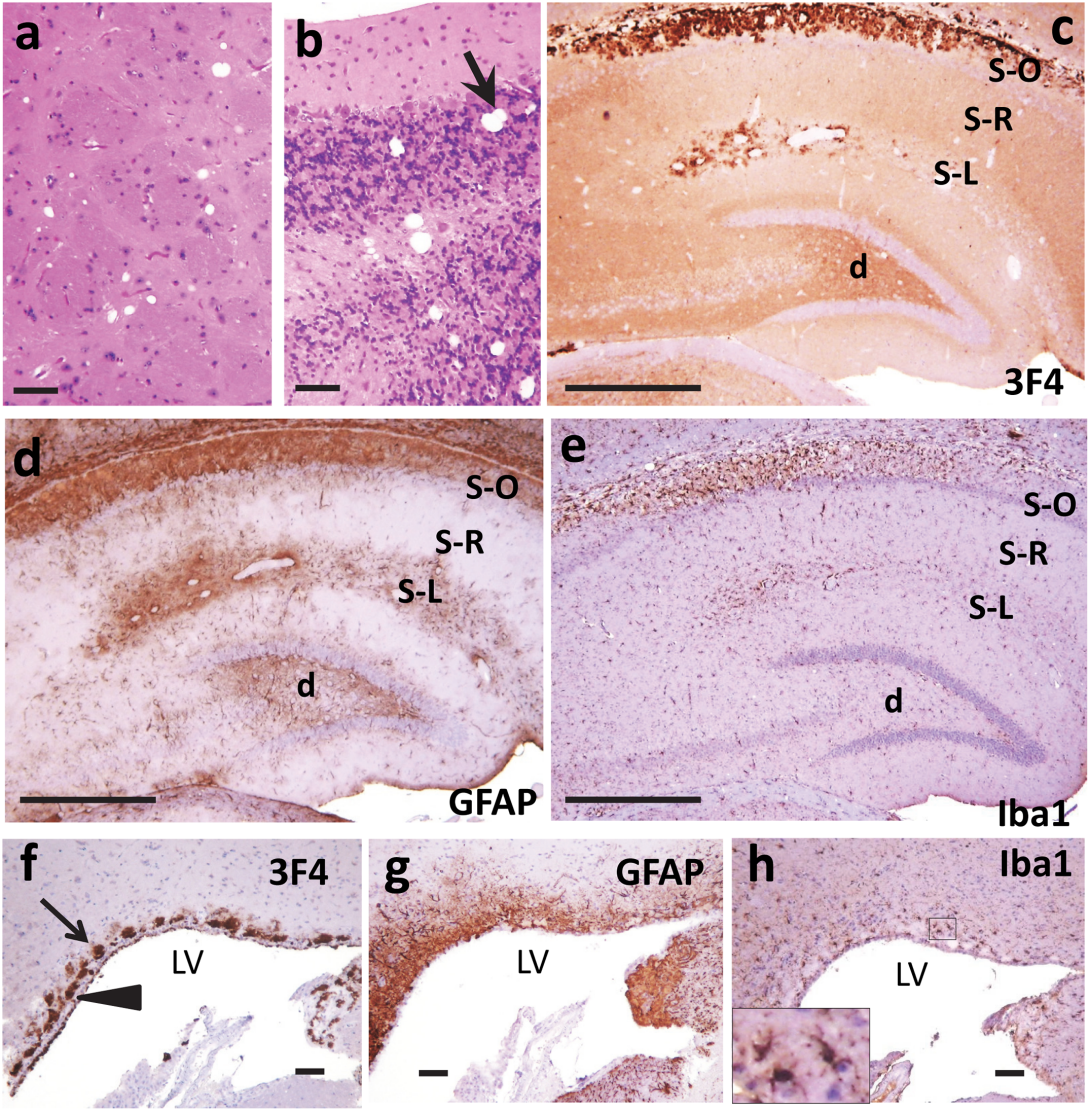
Histopathological analysis of brains from the 2^nd^ passage of PrPres produced in dgPMCAb reactions seeded with fibrillar WT α-synuclein. Representative images of caudate putamen (**a**) and cerebellum (**b**) stained with hematoxylin and eosin, hippocampus stained with anti-PrP 3F4 (**c**), anti-GFAP (**d**) or anti-Iba1 antibody (**e**), or subventricular zones stained with anti-PrP 3F4 (**f**), anti-GFAP (**g**) or anti-Iba1 antibody (**h)**. Subventricular deposits are indicated by arrows and the ventricular surface of ependymal cells is indicated by an arrowhead. S-O, stratum oriens; S-R, stratum radiatum; S-L, stratum lacunosum-moleculare; d, dentate gyrus, LV, lateral ventricle. Scale bars: in **a, b, f, g, h** = 100 μm, **c, d, e** = 500 μm.

**Fig 5:**
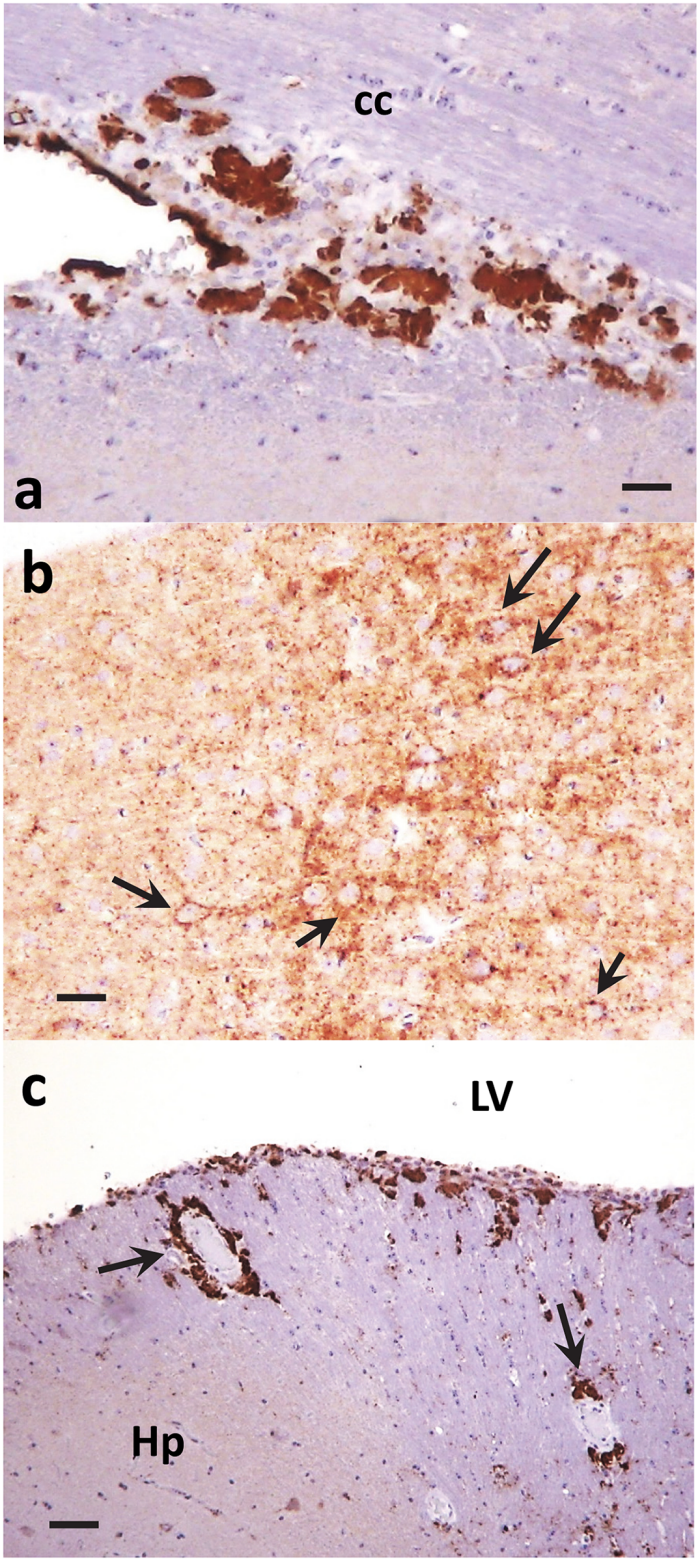
Histopathological analysis of brains from the 2^nd^ passage of PrPres produced in dgPMCAb reactions seeded with fibrillar WT α-synuclein. Subcallosal (**a**), perineuronal (**b**, indicated by arrows) and perivascular (**c**, indicated by arrows) PrP immunoreactivity as stained with anti-PrP 3F4 antibody. Ctx, cortex; cc, corpus callosum; Hp, hippocampus; LV, lateral ventricle. Scale bars: in **a, c** = 100 μm, **b** = 50 μm.

Histopathological examinations of animals with clinical disease from the second passage of dgPMCAb-derived PrPres induced either by α-synuclein WT fibrils or the lysates of HeLa cells expressing α-synuclein A30P revealed features typical for TSE including spongiform degeneration, both reactive astrogliosis and microgliosis, and PrP^Sc^ deposition (Figs 4 and 5). Deposition of PrP immunoreactive with 3F4 was detected in multiple brain regions including cortex, cerebellum, thalamus, hippocampus, and caudate putamen (Figs 4 and 5). Several types of 3F4-positive PrP aggregates were observed, including pronounced plaques and amorphous deposits in the subventricular zones; notable perivascular aggregates, perineuronal deposits and small diffuse deposits in deeper layers of the cortex (Figs 4 and 5). Typically, the areas with PrP deposition showed considerable overlap with the areas of reactive astrogliosis suggesting that astrocytes are activated in the region characterized by PrP replication and/or accumulation (Fig 4b and 4c). Although less pronounced than reactive astrogliosis, the reactive microgliosis was also noticeable (Fig 4d, 4e, 4g and 4h). Similarly to the control animal groups from the first passage, the control group from the 2^nd^ passage of non-seeded dgPMCAb-derived material showed only minor GFAP staining, no significant vacuolization, lack of reactive microgliosis, and lack of PrP^Sc^ deposits immunoreactive with 3F4 (S9 Fig).

## Discussion

The current work demonstrates that aggregated forms of α-synuclein can cross-seed aggregation of the prion protein. Cross-seeding gave rise to PK-resistant, self-replicating PrP states referred to as PrPres that can lead to transmissible prion disease when inoculated and serially passaged in wild type animals. We do not know whether a similar cross-seeding mechanism might take place *in vivo*. When animals were inoculated with fibrillar α-synuclein directly, no clinical, histopathological or biochemical signs of prion disease such as presence of PrPres or PrP^Sc^ as judged by Western blot were observed. The failure to induce prion disease in animals directly by fibrillar α-synuclein could be due to the very low efficiency of cross-seeding *in vivo*, efficient seed clearance and/or prolonged incubation times that exceed the animal’s life expectancy. If this is the case, a much larger cohort of animals would have to be tested to detect possible rare cases of cross-seeding by α-synuclein than the small groups used in the current study.

The format of dgPMCAb assay employed in the current study consisted of seven serial rounds. This amplification protocol was designed to identify fibril preparations capable of seeding prion replication regardless of the amounts of potent seeds present. This format does not intend to compare the relative potency of different fibril preparations, because even miniscule amounts of PrPres could be amplified to the levels detectible by Western blot in seven serial rounds [27,31]. The fact that PrPres was observed only by the fifth dgPMCAb round suggests that the number of active seeds of aggregated α-synuclein capable of initiating PrPres replication is very small and/or that the efficiency of the cross-seeding process is relatively low. Additionally, because α-synuclein forms a variety of aggregated states, including structurally diverse oligomers and fibrils, we do not know the specific α-synuclein states involved in cross-seeding of PrPres [32–34]. We also do not know whether successful seeding by the lysates of cells expressing A30P variant was due to quantitative differences in the amount of aggregates or qualitative differences in the type of aggregates formed in the cells expressing A30P variant versus WT α-synuclein. As judged from experiments performed *in vitro*, while the A30P variant is less fibrillogenic than WT α-synuclein, it is prone to form oligomers and fibrils structurally different from those of WT α-synuclein [34–36]. Moreover, the possibility that misfolded monomers of the A30P variant are capable of cross-seeding should not be completely excluded.

The dgPMCAb substrate used in the current study was PrP^C^ that was treated with PNGase F, which partially removed N-linked glycans from PrP^C^ molecules, changing the ratios of glycoforms in favor of mono- and unglycosylated PrP^C^ at the expense of diglycosylated PrP^C^. Because of this PNGase F treatment, dgPMCAb should be considered as a very artificial *in vitro* system. Previously we showed that changing the glycoform ratios in favor of mono- and unglysocylated PrP^C^ releases structural constraints imposed by N-linked glycans and opens up multiple misfolding pathways, resulting in alternative self-propagating structures including PrPres [27,37]. Even a modest change in the glycoform ratio in favor of mono- and unglysocylated PrP^C^ was sufficient to expand the range of plausible self-replicating PrP structures. As such, in contrast to PMCA, dgPMCAb conditions offer advantages in the search of self-replicating states of amyloidogenic proteins capable of successful cross-seeding of PrP. PrPres characterized by a short, C-terminal, PK-resistant region was found to be the first product of PrP^C^ misfolding initiated by cross-seeding. While the conditions of dgPMCAb are considered artificial, dgPMCAb-derived PrPres was very similar to the C-terminal, PK-resistant fragments observed in the majority of patients with sporadic CJD [38] or in atypical bovine spongiform encephalopathy (H-BSE), which is believed to be sporadic in origin [39]. Analysis of PK-resistant species in brains of sporadic CJD-affected individuals identified PrP-derived fragments corresponding to the C-terminal regions encompassing residues ~154/156-231 and 162/167-231, in addition to PrP^Sc^ [38]. The relationship between *bona fide* PrP^Sc^ and the C-terminal PK-resistant fragments is not clear [40]. Nevertheless, considering that in PrP^Sc^ the C-terminal region is the one that is the most resistant to solvent-induced denaturation [41], the C-terminal PK-resistant fragments found in sporadic CJD might represent an intermediate state toward PrP^Sc^.

While dgPMCAb conditions might be considered non-physiological, the products might still be relevant since the relative expression of PrP^C^ glycoforms in a brain varies in a region-specific manner [42]. Therefore, PNGase F-treated PrP^C^ might represent PrP^C^ in those brain regions that display higher proportions of mono- and un- versus diglycosylated PrP^C^. Remarkably, the PrPres generated in dgPMCAb under conditions with altered PrP^C^ glycoform ratios was capable of propagating in animals despite the high proportion of di- versus mono- and unglycosylated PrP^C^ in brain (Fig 2). This result, along with a fact that PrPres was not amplifiable in PMCAb with non-treated substrate [27], suggests that in brain, PrPres might specifically target only those brain regions or cell types that have a higher proportion of un- and mono-glycosylated PrP^C^. Nevertheless, overall unfavorable ratios of PrP^C^ glycoforms in brain were likely to be responsible for the slow rate of PrPres replication as well as a modest shift in glycoform ratios observed in animal-derived PrPres relative to dgPMCAb-derived PrPres (Fig 2b).

Previous studies on the evolution of synthetic strains helped to establish a relationship between PrPres and PrP^Sc^ [26,28,43,44]. The dynamics between PrPres and PrP^Sc^ described in the current study resembled the changes in self-propagating PrP states during evolution of prion strains of synthetic origin [26,28,43,44]. In previous studies, transmissible prion disease could be produced in wild type animals by inoculating with either rPrP amyloid fibrils or dgPMCAb-derived PrPres formed upon seeding with rPrP amyloid fibrils, and subsequent serial passaging [26,28,43]. PrPres was found to be the first product of PrP^C^ misfolding in animals inoculated with either rPrP amyloid fibrils or dgPMCAb-derived PrPres [26,28,43,44]. In the course of serial transmission, PrPres gave rise to *bona fide* PrP^Sc^ and was replaced by PrP^Sc^ by the end of 2^nd^ or 3^d^ passages [26,28,43,44]. As judged from biochemical assays, PrPres and PrP^Sc^ were structurally different [26,27,40]. Nevertheless, a detailed analysis of the dynamics of the PrPres-to-PrP^Sc^ transition suggested a mechanism, in which PrP^Sc^ forms as a result of rare deformed templating events during replication of PrPres. Once the first PrP^Sc^ particles are generated, PrP^Sc^ replicates independently of PrPres and replaces PrPres because of its faster replication rate [26,43,44]. Similarities in the dynamics between PrPres and PrP^Sc^ in the current work and those observed during evolution of synthetic prions suggest that PrP^Sc^ evolved from transmissible, self-replicating PrPres, which was the first misfolded state triggered by cross-seeding.

Animals from both groups inoculated with dgPMCAb-derived PrPres showed fine diffuse synaptic PrP deposits immunoreactive with SAF-84 antibody in the cortex and hippocampus, but relatively minor astrocytic gliosis and minimal if any spongiosis or microgliosis. Lack of substantial lesions in the first passage despite deposition of considerable amounts of PrPres correlated well with the lack of clinical symptoms and suggested that PrPres is not toxic *per se* and does not lead to inflammation of glia. These results are in good agreement with the previous studies that documented lack of clinical symptoms and neuronal toxicity in animals that had deposits of self-propagating C-terminal PrPres states in the absence of PrP^Sc^ [43–45]. Animals of the second passage displayed clinical symptoms and pronounced lesions including spongiosis, astrocytic gliosis and microgliosis, the major histopathological hallmarks of TSEs. The presence of clinical symptoms and TSE-specific lesions correlated well with accumulation of PrP^Sc^ detectible by immunostaining of brain slices and in Western blots. Several types of 3F4-positive PrP deposits were observed including large plaques in subventricular zones, consistent perivascular aggregates, perineuronal deposits and small diffuse deposits.

One of the most intriguing findings of the current work is that self-replicating PrP states that lead to transmissible prion diseases could arise via cross-seeding by α-synuclein that has no sequence homology with the mammalian prion protein (S3 Fig). Surprisingly, fibrils prepared from mouse rPrP, which is 94% identical to Syrian hamster PrP, did not have any detectible seeding effects with respect to hamster PrP as a substrate in this assay. Moreover, our previous studies demonstrated that Syrian hamster rPrP fibrils (referred to as S fibrils), which had a cross-β folding pattern different from that of the hamster fibrils used in the current work [46,47], also failed to seed PrPres in dgPMCAb conducted in Syrian hamster brain homogenates [26]. Together, these results suggest that commonality in folding patterns of fibrils rather than high sequence identity or homology between seeds and a substrate might be critical for successful cross-seeding.

In the current work, human α-synuclein aggregates were found to cross-seed hamster PrP. Because human α-synuclein and PrP have no sequence homology, regardless of the species-specific sequence of PrP, it is likely that seeding specificity by human α-synuclein is not limited to hamster PrP. Nevertheless, the question of whether the cross-seeding is due to an idiosyncrasy of these two specific players or whether this effect could be generalized is of great clinical importance and should be addressed in future studies. Notably, studies on cross-species prion transmission demonstrated that differences in PrP primary structures between host and donor do not always guarantee a strong species barrier [48]. For instance, prions from a variety of species can be transmitted very effectively to the bank vole despite differences in amino acid sequences, showing very little if any species barrier and suggesting that the bank vole is a universal host [48,49]. On other hand, in certain lines of transgenic mice expressing human PrP^C^ the transmission of a new variant CJD showed significant barrier, as judged from long incubation times, incomplete attack rates or lack of clinical diseases, despite identity in amino acid sequences of the host PrP^C^ and donor PrP^Sc^ [50,51]. The current study, along with the observations that spontaneous non-seeded conversion or conversions seeded with Mo rPrP or Aβ fibrils have not been observed, argues that in dgPMCAb conditions conversion of PrP^C^ into self-propagating PrPres displays a high energy activation barrier. What characteristics of seeds are important for successful cross-seeding? Lack of detectable cross-seeding effects by non-fibrillar α-synuclein or fibrillar Aβ suggests that having amyloid-specific, cross-β folding patterns might be important but is not sufficient for cross-seeding. Consistent with the mechanism postulated by deformed templating [52,53], we propose that successful cross-seeding requires a partial overlap between the cross-β folding pattern of the seed and the folding pattern favored by the primary structure of the substrate, whereas high sequence homology between two proteins is not as important. As bulky N-linked glycans limit the range of possible self-replicating states accessible to PrP^C^ due to spatial interference, their partial cleavage makes the states, that are otherwise prohibited, accessible to PrP^C^.

Although Aβ fibrils formed *in vitro* did not seed formation of PrPres in dgPMCAb, the possibility of prion cross-seeding by Aβ cannot be completely dismissed. Several conformationally distinct strains of Aβ fibrils have been identified in Alzheimer’s disease patients and mouse models of Alzheimer’s disease [54–57]. Moreover, unless seeded with brain extracts from individuals with Alzheimer’s disease, the fibrils produced *in vitro* were found to be structurally different from those formed in human brain [58]. Since it is highly unlikely that our *in vitro* preparations of Aβ fibrils contain structures similar to those formed in human brain, the possibility of cross-seeding of prion formation by naturally occurring Aβ fibrils needs to be examined. Recent studies demonstrated cross-seeding activity of Tau aggregation by Aβ fibrils in cellular assays, producing potent Tau seeds that induced Tau pathology *in vivo* [23]. In support of the hypothesis that the structure of seeds is important in determining the effectiveness of the cross-seeding, two distinct strains of recombinant α-synuclein fibrils produced *in vitro* were shown to cross-seed tau aggregation in primary neurons and transgenic animals with strikingly different efficiency [24]. Nevertheless, the experiment using Aβ fibrils illustrates that not every fibrillar state possesses cross-seeding activity, documenting the high selectivity of cross-seeding under dgPMCAb conditions.

The possibility of cross-seeding raises questions regarding the etiology of prion diseases that are considered to be sporadic in origin. As shown in the current study, cross-seeding might give rise to self-replicating PrP states, which are not toxic *per se* but lead to PrP^Sc^ and prion disease upon serial transmission. One might hypothesize that in patients with α-synuclein pathology, cross-seeding might trigger PrP^C^ misfolding leading to a progression and combined Parkinson’s and prion diseases. Keeping in mind that Parkinson’s disease is more prevalent than sCJD and that the progression of the clinical stage of Parkinson’s disease is slower than that of sCJD, one cannot exclude the possibility that cross-seeding of prions by pathogenic states of α-synuclein might be responsible for a small fraction of sCJD cases. Vice versa, misfolding of PrP^C^ variants associated with genetic prion disease might cross-seed α-synuclein aggregates resulting in mixed brain pathologies. In agreement with this hypothesis, previous studies have described the coexistence of clinical symptoms of CJD and Parkinson’s disease or deposition of both prion and α-synuclein aggregates in brain [3–6,59]. Indeed, 15% of individuals with genetic CJD associated with PrP mutation E200K were found to exhibit Lewy-type α-synuclein pathology [10]. Preclinical multiple system atrophy, which is characterized by inclusions of α-synuclein deposits in glia, was found in a patient who succumbed to sCJD [6]. α-synuclein-immunoreactive deposits have also been found in the central nervous system of patients with various prion diseases, including sCJD, variably protease-sensitive prionopathy, in natural scrapie in sheep and goats, and in hamsters infected with scrapie [4,5,7,12,13].

Which cellular sites are involved in cross-seeding? While the majority of aggregated α-synuclein including α-synuclein A30P mutant is deposited intracellularly in the form of Lewy bodies [60,61] (reviewed in [62]), a series of recent studies described extracellular α-synuclein, which is believed to be responsible for cell-to-cell spread of α-synuclein aggregates [63–67]. Extracellular α-synuclein oligomers were found either in association with exosomes or free [66]. Recent studies suggested that PrP^C^ might be involved in spreading extracellular α-synuclein and that the charged PrP^C^ region encompassing residues 95–110 is responsible for the interaction with α-synuclein [68]. Fibrillar α-synuclein was found to bind strongly to PrP^C^-expressing cells and spread faster in PrP^C^-overexpressing mice in comparison to the wild type or knockout controls [68]. Lysosomes might serve as alternative cellular sites of cross-seeding, as both oligomeric α-synuclein and PrP^C^ are processed through the endo-lysosomal system [67,69–72]. It will be important to establish in future studies whether cross-seeding of prions by α-synuclein occurs *in vivo* and whether it can be induced by extracts of pathological α-synuclein derived from Parkinson’s disease patients. In addition, future studies of the interaction among proteins associated with different neurodegenerative diseases should establish whether the concept of cross-seeding can be generalized.

## Materials and methods

### Ethics statement

This study was carried out in strict accordance with the recommendations in the Guide for the Care and Use of Laboratory Animals of the National Institutes of Health. The animal protocol was approved by the Institutional Animal Care and Use Committee of the University of Maryland, Baltimore (Assurance Number A32000-01; Permit Number: 0215002).

### Expression and purification of rPrP, formation of rPrP fibrils, α-synuclein fibrils and Aβ fibrils

Full-length recombinant mouse or Syrian hamster PrPs (rPrP) were expressed and purified according to a previously described procedure [73]. Lyophilized preparations of rPrPs were dissolved in 5 mM HEPES (pH 7.0) immediately before use. To form mouse rPrP fibrils, the rPrP stock solution was supplemented with 50 mM MES (pH 6.0), three 2/32” Teflon beads (McMaster-Carr, Robbinsville, NJ), and either 0, 0.1, 0.5 or 2.0 M GdnHCl, and incubated at 37°C under continuous agitation. Fibrillation was performed with 0.25 mg/ml rPrP under 600 rpm horizontal shaking using Wallac 1296–004 Delfia Plateshake in a total volume 0.6 ml. Amyloid formation was confirmed by the Thioflavin T (ThT) fluorescence assay as previously described [74] (S1 Table). Syrian hamster rPrP fibrils were formed in 0.5 M GdnHCl as previously described [26].

Human wild type (WT) α-synuclein was from two sources: (#1) purified as previously described [75] and (#2) purchased (cat # S-1001-2, rPeptide, Bogart, GA). The purity of purified α-synuclein was confirmed by electrophoresis on precast 12% SDS-PAGE (S1 Fig). Lyophilized α-synuclein was resuspended in PBS to a final concentration of 280 μM, supplied with 3 2/32” Teflon beads (McMaster-Carr) in a total volume 0.5 ml and incubated at 37°C under 600 rpm horizontal shaking using Wallac 1296–004 Delfia Plateshake. Formation of fibrils was confirmed using the ThT fluorescence assay as described [74]. The fibrils were then used for seeding protein misfolding cyclic amplification with partially deglycosylated substrate (dgPMCAb) or inoculation into Syrian hamsters.

Human Aβ (residues 1–40) was purchased (Pepnome Limited, Hong Kong, China) and subjected to fibrillation in a total volume 0.5 ml under six solvent conditions: (#1) 0.5 mM Aβ, 150 mM HEPES pH 7.4, 150 mM NaCl, 24 h at room temperature; (#2) 0.1 mM Aβ, 10 mM PBS pH 7.4, 7 days at 4°C; (#3): 25 μM Aβ, PBS pH 7.4, 7 days at 25°C; (#4) 50 μM Aβ, 100 mM NaCl, PBS pH 7.4, 24 h at 37°C; (#5): 40 μM Aβ, 0.5 M Tris pH 7.5, 7 days at 37°C with 3 beads (McMaster-Carr); and (#6) 200 μM Aβ, 0.5 M Tris pH 7.5, 7 days at 37°C with 3 beads (McMaster-Carr). For conditions #5 and #6, the reactions were conducted under 600 rpm horizontal shaking using Wallac 1296–004 Delfia Plateshake. Formation of fibrils was confirmed using the ThT fluorescence assay as described [74] (S1 Table). The fibrils were then used as seeds in dgPMCAb (S1 Table).

### Culturing HeLa cells expressing human α-synuclein WT and variant

HeLa cells (American Type Culture Collection, Manassas, VA) were transfected with GFP-tagged human α-synuclein WT or the A30P mutant, and stable cell lines expressing the proteins were isolated. Cells were cultured in Dulbecco’s modified Eagle’s medium (DMEM, Invitrogen) supplemented with 10% fetal bovine serum in the absence of sodium arsenate (cat # 10437, Life Technologies, condition #1), or in the presence of 5 μM sodium arsenate (Sigma-Aldrich) added 1 hour prior to cell harvesting (condition #2). Fluorescent images of live cells were captured using an inverted microscope (Nikon Eclipse TE2000-U) equipped with an illumination system X-cite 120 (EXFO Photonics Solutions Inc, Exton, PA, USA) and a cooled 12-bit CoolSmap HQ CCD camera (Photometrics, Tucson, AZ, USA). Images were processed using WCIF ImageJ software (National Institute of Health, Bethesda, MD, USA).

### Protein misfolding cyclic amplification with beads and partially deglycosylated substrate (dgPMCAb)

10% normal brain homogenate (NBH) from healthy hamsters was prepared as described previously [28]. To produce substrate for dgPMCAb, 10% NBH from healthy hamsters was treated with peptide-N-glycosidase F (PNGase F) (cat # P0705S, New England BioLabs, Ipswich, MA) as follows. After preclearance of NBH at 500 × g for 2 min, 1500 U/ml PNGase F was added to the supernatant, and the reaction was incubated on a rotator at 37°C for 5 h. For serial dgPMCAb reactions, 90 ul PNGase F-treated NBH aliquots were placed in thin-wall PCR tubes, supplied with two 2/32” Teflon beads (McMaster-Carr) [76], and seeded with 10 ul of one of the following: (i) *in vitro*-produced α-synuclein fibrils; (ii) non-fibrillar α-synuclein; (iii) *in vitro*-produced Aβ fibrils; (iv) *in vitro*-produced mouse rPrP fibrils, (v) lysates of HeLa cells expressing wild type (WT) human α-synuclein, or (vi) lysates of HeLa cells expressing A30P variant α-synuclein. As negative controls in each experiment non-seeded dgPMCAb reactions were placed onto the same microplate horn and sonicated/incubated together with seeded dgPMCAb reactions. dgPMCAb reactions were carried out using a Misonix S-4000 microplate horn (Qsonica LLC, Newtown, CT) for seven rounds. Each round consisted of 30 sec sonication pulses delivered at 170W energy output applied every 30 min during a 24 hour period. For each subsequent round, 10 μl of the reaction from the previous round were added to 90 μl of fresh substrate.

### Proteinase K digestion assay of dgPMCAb-derived products

Ten μl of dgPMCAb-derived samples were supplemented with 5 μl SDS and 5 μl PK to a final concentration of 0.25% SDS and 25 μg/ml PK and incubation for 1 hour at 37°C. The digestion was terminated by addition of SDS sample buffer and heating the samples for 10 min in a boiling water bath. Samples were loaded onto NuPAGE 12% BisTris gels, transferred to PVDF membrane, and probed with anti-PrP SAF-84 antibodies (Cayman Chemical, Ann Arbor, MI).

### Animal bioassay

Four to five week old Golden Syrian hamsters (all males, Harlan Laboratories, Indianapolis, IN) were inoculated intracranically (IC) into the left hemisphere, ~3 mm to the left of the midline and ~3 mm anterior to a line drawn between the ears under 2% isoflurane anesthesia. Each animal received 50 μl of dgPMCAb-derived materials diluted 10-fold in 1% BSA/PBS or 50 μl of α-synuclein (280 μM in a fibrillar or non-fibrillar state) diluted 10-fold in PBS (final concentration in inocula 28 μM). After inoculation, hamsters were observed daily for disease using a ‘blind’ scoring protocol. Animals from the first passage did not develop any clinical symptoms and were euthanized at 561 days post inoculation by asphyxiation with CO_2_ (Table 1). For the second passage, 10% brain homogenates (BH) in PBS were dispersed by 30 sec of sonication immediately before inoculation. Each hamster received 50 μl of 10% BH inoculum IC under 2% isoflurane anesthesia and was observed daily for disease using a ‘blind’ scoring protocol. The following symptoms were observed: a non-habituating startle response to sound and touch; an agitated, fidgeting behavior; dry skin, patchy and shedding hair. Animals were sacrificed at 638–642 days post inoculation by asphyxiation with CO_2_ (Table 1). Animals that did not develop clinical signs of disease were euthanized at 642 days post inoculation (Table 1). Animals were not perfused.

### Proteinase K digestion assay of brain-derived products

10% (wt/vol) brain homogenate (BH) was prepared in PBS, pH 7.4, using glass/Teflon homogenizers attached to a cordless 12 V compact drill (Ryobi) as previously described [77]. 10% BH was briefly sonicated and mixed with an equal volume of 4% sarcosyl in PBS, supplemented with 50 mM Tris, pH 7.5, and digested with 20 μg/ml PK (cat # P8107S, New England BioLabs, Ipswich, MA) for 30 min at 37°C with shaking. PK digestion was stopped by adding SDS sample buffer and heating the samples for 10 min in a boiling water bath. Samples were loaded onto NuPAGE 12% Bis-Tris gels, transferred to PVDF membranes, and probed with 3F4 or SAF-84 antibodies.

### Histopathological studies

Histopathological studies were performed on three animals per group. Formalin fixed brain halves were divided at the midline. Right hemisphere was frozen, and left hemisphere was fixed in 10% neutral buffered formalin solution. Formalin-fixed hemispheres were paraffin embedded, sliced into 4 μm sections and processed for hematoxylin-eosin stain as well as for immunohistochemistry for PrP using the mouse monoclonal anti-PrP antibody SAF-84 (1:1000, Cayman Chemical) and 3F4 (1:1000, Covance, Berkeley, CA, USA), anti-glial fibrillar acidic protein (GFAP; 1:3000, Dako, Glostrup, Denmark), or anti-Iba1 antibody (1:500, Wako, Richmond, VA, USA). Horse radish peroxidase-labeled goat anti-rabbit and anti-mouse antibody (KPL, Milford, MA) were used as secondary antibody for GFAP and Iba1 (rabbit), 3F4 (mouse) and SAF-84 (mouse). Detection was performed using DAB Quanto chromogen and substrate (VWR, Radnor, PA). Brains were treated in formic acid (96%) prior to embedding in paraffin to deactivate prion infectivity. For detection of disease-associated PrP, we applied a pretreatment of 30 minutes hydrated autoclaving at 121°C followed by 5 minutes in 96% formic acid. We evaluated the brain for the presence of inflammatory infiltrates, spongiform changes, degree of gliosis, and PrP immunoreactivity.

For staining of α-synuclein, brain sections were pretreated by microwaving for 10 minutes in citrate buffer (pH 6), followed by treatment in 98% formic acid for 1 minute. Immunostainings was performed using anti-α-synuclein antibodies: 4D6 (1:10,000, immunogen: human alpha-Synuclein; Covance, Emeryville, CA, USA).

## Supporting information

S1 Table Fibril formation for Aβ, Mo rPrP and α-synuclein as monitored by ThT fluorescence assay. (DOCX)

S1 Fig Estimation of purity of purified α-synuclein using electrophoresis on precast 12% Bis-Tris SDS-PAGE followed by staining with Coomassie Blue. (PDF)

S2 Fig Fluorescence microscopy imaging of HeLa cells expressing human WT α-synuclein (**a**) or A30P variant α-synuclein (**b**) detected by GFP fluorescence. Fluorescence microscopy imaging of amyloid fibrils prepared *in vitro* using human WT α-synuclein (**c**) or Aβ peptide (**d**) and stained with Thioflavin T. Thioflavin T staining and microscopy imaging of amyloid fibrils was performed as described earlier [74]. Scale bars = 5 μm.
(PDF)

S3 Fig Amino acid sequences of human, Syrian hamster and mouse prion proteins and human α-synuclein. (PDF)

S4 Fig Histopathological analysis of brains from the 1^st^ passage of dgPMCAb-derived PrPres. Representative images of the thalamus of animals inoculated with dgPMCAb products seeded with fibrillary α-synuclein (**a, b**) or dgPMCAb products seeded with lysates of HeLa cells expressing A30P α-synuclein (**c, d**). Note the lack of spongiform change in the sections stained with hematoxylin and eosin (**a, c**). Immunostaining for PrP using SAF-84 (**b, d**) revealed diffuse/synaptic and granular deposits. Scale bar in a = 50 μm.
(PDF)

S5 Fig Histopathological analysis of brains from the 1^st^ passage of dgPMCAb-derived PrPres. Representative images of the frontal cortex (**a, c**) and hippocampus (**b, d**) of animals inoculated with dgPMCAb products seeded with fibrillary α-synuclein (**a, b**) or dgPMCAb products seeded with lysates of HeLa cells expressing A30P α-synuclein (**c, d**) and stained with 3F4 antibody. Scale bar in **a** = 50 μm.
(PDF)

S6 Fig Histopathological analysis of microglia in brains from the 1^st^ passage of dgPMCAb-derived PrPres stained for Iba1. Representative images of the frontal cortex (**a, d**), hippocampus (**b, e**) and thalamus (**c, f**) of animals inoculated with dgPMCAb products seeded with fibrillar WT α-synuclein (**a-c**) or dgPMCAb products seeded with lysates of HeLa cells expressing A30P α-synuclein (**d-f**). Scale bar in **a** = 50 μm.
(PDF)

S7 Fig Histopathological analysis of brains of animals inoculated with products of non-seeded dgPMCAb reactions (a-h), WT α-synuclein fibrils (i-p), or non-fibriallar α-synuclein (q-x). Representative images of the frontal cortex (**a-d, i-l, q-t**) and hippocampus (**e-h, m-p, u-x**) stained with hematoxylin and eosin (**a, d, g, j, m, p**), anti-PrP SAF-84 antibody (**b, f, j, n, r, v**), anti-GFAP antibody (**c, g, k, o, s, w**) or anti-Iba1 antibody (**d, h, l, p, t, x**) Scale bar = 100 μm.
(PDF)

S8 Fig Histopathological analysis for α-synuclein in Syrian hamsters inoculated with dgPMCAb products seeded with fibrillary WT α-synuclein (a-c) and un-inoculated age-matched controls (d-f). Representative images of the hippocampus (**a, d**), caudate-putamen (**b, e**) and cerebellum (**c, f**) showing the physiological synaptic immunostaining for α-synuclein using the 4D6 antibody. Three animals from each group were examined. Scale bar in **a** = 50 μm for **a-c** and 25 μm for **d-f**.
(PDF)

S9 Fig Histopathological analysis of brains from the 2^d^ passage of dgPMCAb-derived material produced in non-seeded reactions. Representative images of caudate putamen (a) and cerebellum (b) stained with hematoxylin and eosin, hippocampus stained with anti-PrP 3F4 (c), anti-GFAP (d) or anti-Iba1 antibody (e), or subventricular zones stained with anti-PrP 3F4 (f), anti-GFAP (g) or anti-Iba1 antibody (h). S-O, stratum orients; S-R, stratum radiatum; S-L, stratum lacunosum-moleculare; d, dentate gyrus, LV, lateral ventricle. Scale bars: in **a, b, f, g, h** = 100 μm, **c, d, e** = 500 μm.
(PDF)

## References

[1] Prusiner SB (2001) Shattuck Lecture—Neurodegenerative diseases and prions. NEnglJMed 344: 1516–1526. 10.1056/NEJM200105173442006 11357156

[2] Kovacs GG (2016) Molecular Pathological Classification of Neurodegenerative Diseases: Turning towards Precision Medicine. Int J Mol Sci 2: E189. 10.3390/ijms17020189 PMC4783923 26848654

[3] Iida T, Doh-ura K, Kawashima T, Abe H, Iwaki T (2001) An atypical case of sporadic Creutzfeldt-Jakob disease with Parkinson's disease. Neuropathology 21: 294–297. 11837536 10.1046/j.1440-1789.2001.00407.x

[4] Vital A, Canron M-H, Gil R, Hauw J-J, Vital C (2007) A sporadic case of Creutzfeldt-Jakob disease with beta-amyloid deposits and alpha-synuclein inclusions. Neuropathology 27: 273–277. 17645242 10.1111/j.1440-1789.2007.00755.x

[5] Head MW, Lowrie S, Chohan G, Knight R, Scoones DJ, et al (2010) Variably protease-sensitive prionopathy in a PRNP codon 129 heterozygous UK patient with co-existing tay, α synuclein and Aβ pathology. Acta Neuropath 120: 821–823. doi: 10.1007/s00401-010-0766-y 21046409 10.1007/s00401-010-0766-y

[6] Rodriguez-Diehl R, Rey MJ, Gironell A, Martinez-Saez E, Ferrer I, et al (2012) "Preclinical" MSA in definite Creutzfeldt-Jakob disease. Neuropathology 32: 158–163. doi: 10.1111/j.1440-1789.2011.01232.x 21692862 10.1111/j.1440-1789.2011.01232.x

[7] Vital A, Fernagut P-O, Canron M-H, Joux J, Bezard E, et al (2009) The nigrostriatal pathway in Creutzfeldt-Jakob disease. J Neuropathol Exp Neurol 68: 809–815. doi: 10.1097/NEN.0b013e3181abdae8 19535991 10.1097/NEN.0b013e3181abdae8

[8] Tschampa HJ, Neumann M, Zerr I, Henkel K, Schroter A, et al (2001) Patients with Alzheimer's disease and dementia with Lewy bodies mistaken for Creutzfeldt-Jakob disease. J Neurol Neurosurg Psychiatry 71: 33–39. doi: 10.1136/jnnp.71.1.33 11413259 10.1136/jnnp.71.1.33 PMC1737446

[9] Rahimi J, Kovacs GG (2014) Prevalence of mixed pathologies in the aging brain. Alzheimers Res Ther 6: e82. 10.1186/s13195-014-0082-1 PMC4239398 25419243

[10] Kovacs GG, Seguin J, Quadrio I, Hoftberger R, Kapas I, et al (2011) Genetic Creutzfeldt-Jakob disease associated with the E200K mutation: characterization of a complex proteinopathy. Acta Neuropath 121: 39–57. doi: 10.1007/s00401-010-0713-y 20593190 10.1007/s00401-010-0713-y

[11] Haik S, Brandel JP, Sazdovitch V, Delasnerie-Laupretre N, Peoc'h K, et al (2000) Dementia with Lewy bodies in a neuropathologic series of suspected Creutzfeldt-Jakob disease. Neurology 55: 1401–1404. 11087793 10.1212/wnl.55.9.1401

[12] Haik S, Privat N, Adjou KT, Sazdovitch V, Dormont D, et al (2002) Alpha-synuclein-immunoreactive deposits in human and animal prion diseases. Acta Neuropath(Berlin) 103: 516–520. 11935269 10.1007/s00401-001-0499-z

[13] Adjou KT, Allix S, Ouidja MO, Backer S, Couquet C, et al (2007) Alpha-synuclein accumulates in the brain of scrapie-affected sheep and goats. JCompPathol 137: 78–81. 10.1016/j.jcpa.2007.03.007 17544436

[14] Miyazono M, Kitamoto T, Iwaki T, Tateishi J (1992) Colocalization of prion protein and beta protein in the same amyloid plaques in patients with Gerstmann-Straussler syndrom. Acta Neuropathol 83: 333–339. 1349451 10.1007/BF00713522

[15] Takahashi M, Hoshii Y, Kawano H, Gondo T, Ishihara T, et al (1996) Ulstrastructural evidence for collocalization of kappa light chain- and beta 2-microglobulin -derived amyloid using double labelling immunogold electron microscopy. Virchows Arch 429: 383–388. 8982384 10.1007/BF00198444

[16] Galuske RA, Drach LM, Nichtweiss M, Marquardt G, Franz K, et al (2004) Colocalization of different types of amyloid in the walls of cerebral blood vessels of patients from cerebral amyloid angiopathy and spontaneous intracranial hemorrhage: a report of 5 cases. ClinNeuropathol 23: 113–119. 15200289

[17] Kovacs GG, Rahimi J, Strobel T, Lutz MI, Regelsberger G, et al (2017) Tau Pathology in Creutzfeldt-Jakob Disease Revisited. Brain Pathol 27: 332–344. doi: 10.1111/bpa.12411 27377321 10.1111/bpa.12411 PMC8028936

[18] Mougenot AL, Bencsik A, Nicot S, Vulin J, Morigant E, et al (2011) Transmission of prion strains in a transgenic mouse model overexpressing human A53T mutated α-synuclein. J Neuropathol Exp Neurol 70: 377–385. doi: 10.1097/NEN.0b013e318217d95f 21487306 10.1097/NEN.0b013e318217d95f

[19] Masliah E, Rockenstein E, Inglis C, Adame A, Bett C, et al (2012) Prion infection promotes extensive accumulation of α-synuclein in aged human α-synuclein transgenic mice. Prion 6: 184–190. doi: 10.4161/pri.19806 22460692 10.4161/pri.19806 PMC3366356

[20] Derkatch IL, Bradley ME, Hong JY, Liebman SW (2001) Prions affect the appearance of other prions: the story of [PIN(+)]. Cell 106: 171–182. 11511345 10.1016/s0092-8674(01)00427-5

[21] Derkatch IL, Uptain SM, Quteiro TF, Krishnan R, Lindquist SL, et al (2004) Effects of Q/N-rich, polyQ, and non-polyQ, amyloids on the de novo formation of the [PSI+] prion in yeast and aggregation of Sup35 in vitro. ProcAcadNatlSciUSA 101: 12934–12939. 10.1073/pnas.0404968101 PMC516497 15326312

[22] Yan J, Fu X, Ge F, Zhang B, Yao J, et al (2007) Cross-seeding and cross-competition in mouse apolipoprotein A-II amyloid fibrils and protein A amyloid fibrils. American Journal of Pathology 171: 172–180. doi: 10.2353/ajpath.2007.060576 17591964 10.2353/ajpath.2007.060576 PMC1941612

[23] Vasconcelos B, Stancu IC, Buist A, Bird M, Wang P, et al (2016) Heterotypic seeding of Tau fibrillization by pre-aggregated Abeta provides potent seeds for prion-like seeding and propagation of Tau-pathology in vivo. Acta Neuropath 131: 549–569. doi: 10.1007/s00401-015-1525-x 26739002 10.1007/s00401-015-1525-x PMC4789256

[24] Guo JL, Covell DJ, Daniels JP, Iba M, Stieber A, et al (2013) Distinct alpha-synuclein strains differentially promote tau inclusions in neurons. Cell 154: 103–117. doi: 10.1016/j.cell.2013.05.057 23827677 10.1016/j.cell.2013.05.057 PMC3820001

[25] Giasson BI, Forman MS, Higuchi M, Golbe LI, Graves CL, et al (2003) Initiation and synergistic fibrillization of tau and alpha-synuclein. Science 300: 636–640. doi: 10.1126/science.1082324 12714745 10.1126/science.1082324

[26] Makarava N, Kovacs GG, Savtchenko R, Alexeeva I, Ostapchenko VG, et al (2012) A New Mechanism for Transmissible Prion Diseases. J Neurosci 32: 7345–7355. doi: 10.1523/JNEUROSCI.6351-11.2012 22623680 10.1523/JNEUROSCI.6351-11.2012 PMC3368278

[27] Makarava N, Savtchenko R, Baskakov IV (2013) Selective amplification of classical and atypical prions using modified protein misfolding cyclic amplification J Biol Chem 288: 33–41. doi: 10.1074/jbc.M112.419531 23168413 10.1074/jbc.M112.419531 PMC3537030

[28] Makarava N, Kovacs GG, Savtchenko R, Alexeeva I, Budka H, et al (2011) Genesis of mammalian prions: from non-infectious amyloid fibrils to a transmissible prion disease. PLoS Pathogen 7: e1002419. 22144901 10.1371/journal.ppat.1002419 PMC3228811

[29] Makarava N, Bocharova OV, Salnikov VV, Breydo L, Anderson M, et al (2006) Dichotomous versus palm-type mechanisms of lateral assembly of amyloid fibrils. ProtScience 15: 1334–1341. 10.1110/ps.052013106 PMC2265092 16731968

[30] Makarava N, Lee CI, Ostapchenko VG, Baskakov IV (2007) Highly promiscuous nature of prion polymerization. JBiolChem 282: 36704–36713. 10.1074/jbc.M704926200 17940285

[31] Makarava N, Savtchenko R, Alexeeva I, Rohwer RG, Baskakov IV (2012) Fast and ultrasensitive method for quantitating prion infectivity titer. Nature Commun 3: 741. 22415832 10.1038/ncomms1730 PMC3518416

[32] Bousset L, Pieri L, Ruiz-Arlandis G, Gath J, Jensen PH, et al (2013) Structural and functional characterization of two alpha-synuclein strains. Nat Commun 4: 2575 doi: 10.1038/ncomms3575 24108358 10.1038/ncomms3575 PMC3826637

[33] Heise H, Hoyer W, Becker S, Andronesi OC, Riedel D, et al (2005) Molecular-level secondary structure, polymorphism, and dynamics of full-length alpha-synuclein fibrils studied by solid-state NMR. Proc Natl Acad Sci USA 102: 15871–15876. doi: 10.1073/pnas.0506109102 16247008 10.1073/pnas.0506109102 PMC1276071

[34] Nielsen SB, Macchi F, Raccosta S, Langkilde AE, Glehm L, et al (2013) Wildtype and A30P Mutant Alpha-Synuclein Form Different Fibril Structures. PLoS One 8: e67713 doi: 10.1371/journal.pone.0067713 23861789 10.1371/journal.pone.0067713 PMC3701545

[35] Flagmeier P, Meisl G, Vendruscolo M, Knowles TPJ, Dobson CM, et al (2016) Mutations associated with familial Parkinson’s disease alter the initiation and amplification steps of α-synuclein aggregation. Proc Natl Acad Sci USA 113: 10328–10333. doi: 10.1073/pnas.1604645113 27573854 10.1073/pnas.1604645113 PMC5027465

[36] Sierecki E, Giles N, Bowden Q, Polinkovsky ME, Steinbeck J, et al (2016) Nanomolar oligomerization and selective co-aggregation of α-synuclein pathogenic mutants revealed by single-molecule fluorescence. Sci Rep doi: 10.1038/srep37630: 37630 27892477 10.1038/srep37630 PMC5385372

[37] Baskakov IV, Katorcha E (2016) Multifaceted role of sialylation in prion diseases. Front Neurosci 10: 358 doi: 10.3389/fnins.2016.00358 27551257 10.3389/fnins.2016.00358 PMC4976111

[38] Zou WQ, Capellari S, Parchi P, Sy MS, Gambetti P, et al (2003) Identification of Novel Proteinase K-resistant C-terminal Fragments of PrP in Creutzfeldt-Jakob Disease. JBiolChem 278: 40429–40436. 10.1074/jbc.M308550200 12917418

[39] Biacabe AG, Jacobs JG, Bencsik A, Langeveld JP, Baron TG (2007) H-type bovine spongiform encephalopathy: complex molecular features and similarities with human prion diseases. Prion 1: 61–68. 19164888 10.4161/pri.1.1.3828 PMC2633710

[40] Klimova N, Makarava N, Baskakov IV (2015) The diversity and relationship of prion protein self-replicating states. Virus Research 207: 113–119. doi: 10.1016/j.virusres.2014.10.002 25312451 10.1016/j.virusres.2014.10.002 PMC4395513

[41] Kocisko DA, Lansbury JPT, Caughey B (1996) Partial unfolding and refolding of scrapie-associated prion protein: evidence for a critical 16-kDa C-terminal domain. Biochemistry 35: 13434–13442. doi: 10.1021/bi9610562 8873612 10.1021/bi9610562

[42] Beringue V, Mallinson G, Kaisar M, Tayebi M, Sattar Z, et al (2003) Regional heterogeneity of cellular prion protein isoforms in the mouse brain. Brain 126: 2065–2073. doi: 10.1093/brain/awg205 12821516 10.1093/brain/awg205

[43] Makarava N, Savtchenko R, Baskakov IV (2015) Two alternative pathways for generating transmissible prion disease de novo. Acta Neuropathologica Communications 3: 69 doi: 10.1186/s40478-015-0248-5 26556038 10.1186/s40478-015-0248-5 PMC4641408

[44] Makarava N, Savtchenko R, Alexeeva I, Rohwer RG, Baskakov IV (2016) New Molecular Insight into Mechanism of Evolution of Mammalian Synthetic Prions. Am J Pathol 186: 1006–1014. doi: 10.1016/j.ajpath.2015.11.013 26873446 10.1016/j.ajpath.2015.11.013 PMC5848243

[45] Kovacs GG, Makarava N, Savtchenko R, Baskakov IV (2013) Atypical and classical forms of the disease-associated state of the prion protein exhibit distinct neuronal tropism, deposition patterns, and lesion profiles. Am J Pathol 183: 1539–1547. doi: 10.1016/j.ajpath.2013.07.024 24012784 10.1016/j.ajpath.2013.07.024 PMC3814526

[46] Makarava N, Baskakov IV (2008) The same primary structure of the prion protein yields two distinct self-propagating states. JBiolChem 283: 15988–15996. 10.1074/jbc.M800562200 PMC2414308 18400757

[47] Ostapchenko VG, Sawaya MR, Makarava N, Savtchenko R, Nilsson KP, et al (2010) Two amyloid states of the prion protein display significantly different folding patterns. JMolBiol 400: 908–921. 10.1016/j.jmb.2010.05.051 PMC2908243 20553730

[48] Nonno R, Di Bari MA, Cardone F, Vaccari G, Fazzi P, et al (2006) Efficient Transmission and Characterization of Creutzfeldt-Jakob Disease Strains in Bank Voles. PLOS Pathog 2: e12 doi: 10.1371/journal.ppat.0020012 16518470 10.1371/journal.ppat.0020012 PMC1383487

[49] Di Bari MA, Nonno R, Castilla J, D'Agostino C, Pirisinu L, et al (2013) Chronic wasting disease in bank voles: characterisation of the shortest incubation time model for prion diseases. PLoS Pathog 9: e1003219 doi: 10.1371/journal.ppat.1003219 23505374 10.1371/journal.ppat.1003219 PMC3591354

[50] Bishop MT, Hart P, Aitchison L, Baybutt HN, Plinston C, et al (2006) Predicting susceptibility and incubation time of human-to-human transmission of vCJD. Lancet Neurol 5: 393–398. doi: 10.1016/S1474-4422(06)70413-6 16632309 10.1016/S1474-4422(06)70413-6

[51] Asante EA, Linehan JM, Desbruslais M, Joiner S, Gowland I, et al (2002) BSE prions propagate as either variant CJD-like or sporadic CJD-like prion stains in transgenic mice expressing human prion protein. EMBO J 21 6358–6366. doi: 10.1093/emboj/cdf653 12456643 10.1093/emboj/cdf653 PMC136957

[52] Makarava N, Baskakov IV (2013) The Evolution of Transmissible Prions: The Role of Deformed Templating. PLOS Pathog 9: e1003759 doi: 10.1371/journal.ppat.1003759 24339773 10.1371/journal.ppat.1003759 PMC3855475

[53] Baskakov IV (2009) Switching in amyloid structure within individual fibrils: implication for strain adaptation, species barrier and strain classification. FEBS Lett 583: 2618–2622. doi: 10.1016/j.febslet.2009.05.044 19482025 10.1016/j.febslet.2009.05.044 PMC2752868

[54] Paravastu AK, Leapman RD, Yau WM, Tycko R (2008) Molecular structural basis for polymorphism in Alzheimer's beta-amyloid fibrils. ProcAcadNatlSciUSA 105: 18349–18354. 10.1073/pnas.0806270105 PMC2587602 19015532

[55] Lu J, Qiang W, Yau WM, Schwieters CD, Meredith SC, et al (2013) Molecular structure of β-amyloid fibrils in Alzheimer's disease brain tissue. Cell 154: 1257–1268. doi: 10.1016/j.cell.2013.08.035 24034249 10.1016/j.cell.2013.08.035 PMC3814033

[56] Stohr J, Condello C, Watts CJ, Bloch L, Oehler A, et al (2014) Distinct synthetic Aβ prion strains producing different amyloid deposits in bigenic mice. Proc Acad Natl Sci U S A 111: 10329–10334. 10.1073/pnas.1408968111 PMC4104853 24982137

[57] Watts CJ, Condello C, Stohr J, Oehler A, Lee J, et al (2014) Serial propagation of distinct strains of Aβ prions from Alzheimer's disease patients. Proc Acad Natl Sci U S A 111: 10323–10328. 10.1073/pnas.1408900111 PMC4104857 24982139

[58] Paravastu AK, Qahwash I, Leapman RD, Meredith SC, Tycko R (2009) Seeded growth of b-amyloid fibrils from Alzheimer's brain-derived fibrils produces a distinct fibril structure. ProcAcadNatlSciUSA 106: 7443–7448. 10.1073/pnas.0812033106 PMC2678625 19376973

[59] Ezrin-Waters C, Resch L, Lang AE (1985) Coexistance of idiopathic Parkinson's disease and Creutzfeldt Jakob disease. Can J Neurol Sci 12: 272–273. 3902185 10.1017/s0317167100047156

[60] Spillantini MG, Schmidt ML, Lee VMY, Trojanowski JQ, Jakes R, et al (1997) à-Synuclein in Lewy bodies. Nature 388: 839–840. doi: 10.1038/42166 9278044 10.1038/42166

[61] Seidel K, Schols L, Nuder S, Petrasch-Parwez E, Gierga K, et al (2010) First Appraisal of Brain Pathology Owing to A30P Mutant Alpha-Synuclein. Ann Neurol 67: 684–689. doi: 10.1002/ana.21966 20437567 10.1002/ana.21966

[62] Braak H, Tredici KD (2017) Neuropathological Staging of Brain Pathology in Sporadic Parkinson’s disease: Separating the Wheat from the Chaff. J Parkinsons Dis 7: S73–S87 28282810 10.3233/JPD-179001 PMC5345633

[63] Desplats P, Lee HJ, Bae EJ, Patrick C, Rockenstein E, et al (2009) Inclusion formation and neuronal cell death through neuron-to-neuron transmission of alpha-synuclein. Proc Natl Acad Sci USA 106: 13010–13015. doi: 10.1073/pnas.0903691106 19651612 10.1073/pnas.0903691106 PMC2722313

[64] Lee HJ, Patel S, Lee SJ (2005) Intravesicular localization and exocytosis of alpha-synuclein and its aggregates. J Neurosci 25: 6016–6024. doi: 10.1523/JNEUROSCI.0692-05.2005 15976091 10.1523/JNEUROSCI.0692-05.2005 PMC6724798

[65] Emmanouilidou E, Melachroinou K, Roumeliotis T, Garbis SD, Ntzouni M, et al (2010) Cell-produced alpha-synuclein is secreted in a calcium-dependent manner by exosomes and impacts neuronal survival. J Neurosci 30: 6838–6851. doi: 10.1523/JNEUROSCI.5699-09.2010 20484626 10.1523/JNEUROSCI.5699-09.2010 PMC3842464

[66] Danzer KM, Kranich LR, Ruf WP, Cagsal-Getkin O, Winslow AR, et al (2012) Exosomal cell-to-cell transmission of alpha synuclein oligomers. Mol Neurodegener 7: 42 doi: 10.1186/1750-1326-7-42 22920859 10.1186/1750-1326-7-42 PMC3483256

[67] Kovacs GG, Breydo L, Green R, Kis V, Puska G, et al (2014) Intracellular processing of disease-associated α-synuclein in the human brain suggests prion-like cell-to-cell spread. Neurobiol Dis 69: 76–92. doi: 10.1016/j.nbd.2014.05.020 24878508 10.1016/j.nbd.2014.05.020

[68] Urrea L, Segura-Feliu M, Masuda-Suzukake M, Hervera A, Pedraz L, et al (2017) Involvement of Cellular Prion Protein in α-Synuclein Transport in Neurons. Mol Neurobiol in press. 10.1007/s12035-017-0451-4 PMC5840251 28229331

[69] Domert J, Sackmann C, Severinsson E, Agholme L, Bergstrom J, et al (2016) Aggregated Alpha-Synuclein Transfer Efficiently between Cultured Human Neuron-Like Cells and Localize to Lysosomes. PLoS One 11: e0168700 doi: 10.1371/journal.pone.0168700 28030591 10.1371/journal.pone.0168700 PMC5193351

[70] Laszlo L, Lowe J, Self T, Kenward N, Landon M, et al (1992) Lysosomes as key organelles in the pathogenesis of prion encephalopathies. JPathol 166: 333–341. 1355530 10.1002/path.1711660404

[71] Taraboulos A, Raeber AJ, Borchelt DR, Serban D, Prusiner SB (1992) Synthesis and trafficking of prion proteins in cultured cells. MolBiolCell 3: 851–863. 10.1091/mbc.3.8.851 PMC275644 1356522

[72] Shyng SL, Huber MT, Harris DA (1993) A prion protein cycles between the cell surface and an endocytic compartment in cultured neuroblastoma cells. JBiolChem 21: 15922–15928. 8101844

[73] Makarava N, Kovacs GG, Bocharova OV, Savtchenko R, Alexeeva I, et al (2010) Recombinant prion protein induces a new transmissible prion disease in wild type animals. Acta Neuropathol 119: 177–187. doi: 10.1007/s00401-009-0633-x 20052481 10.1007/s00401-009-0633-x PMC2808531

[74] Bocharova OV, Breydo L, Parfenov AS, Salnikov VV, Baskakov IV (2005) In vitro conversion of full length mammalian prion protein produces amyloid form with physical property of PrPSc. JMolBiol 346 645–659. 10.1016/j.jmb.2004.11.068 15670611

[75] Jarvela TS, Lam HA, Helwig M, Lorenzen N, Otzen DE, et al (2016) The neural chaperone proSAAS blocks α-synuclein fibrillation and neurotoxicity. Proc Natl Acad Sci USA 113: E4708–E4715. doi: 10.1073/pnas.1601091113 27457957 10.1073/pnas.1601091113 PMC4987805

[76] Gonzalez-Montalban N, Makarava N, Ostapchenko VG, Savtchenko R, Alexeeva I, et al (2011) Highly Efficient Protein Misfolding Cyclic Amplification. PLoS Pathogen 7: e1001277. 21347353 10.1371/journal.ppat.1001277 PMC3037363

[77] Makarava N, Kovacs GG, Savtchenko R, Alexeeva I, Budka H, et al (2012) Stabilization of a prion strain of synthetic origin requires multiple serial passages. J Biol Chem 287: 30205–30214. doi: 10.1074/jbc.M112.392985 22807452 10.1074/jbc.M112.392985 PMC3436274

