# Supplementary material for: Cross-seeding of prions by aggregated α-synuclein leads to transmissible spongiform encephalopathy
Source: PLoS Pathog. 2017 Aug 10;13(8):e1006563. doi: 10.1371/journal.ppat.1006563 (PMC5567908; doi:10.1371/journal.ppat.1006563)
Supplement: S1 Table — (DOCX) [file ppat.1006563.s001.docx]

**Table S1. Fibril formation for Aβ, Mo rPrP and α-synuclein as monitored by ThT fluorescence assay**

|  | Aβ  #1 | Aβ  #2 | Aβ  #3 | Aβ  #4 | Aβ  #5 | Aβ  #6 | Mo rPrP  2M GdnCl | Mo rPrP  0.5M GdnCl | Mo rPrP  0.1M GdnCl | Mo rPrP  0M GdnCl | α-synuclein  source #1 | α-synuclein  source #2 |
| --- | --- | --- | --- | --- | --- | --- | --- | --- | --- | --- | --- | --- |
| 0 hours | 60-65 | | | | | | | | | | | |
| 3^rd^ day | 930^a^ | 114 | 96 | 95 | 180 | 1400 | 187 | 83 | 65 | 67 | 248 | 800 |
| 4^th^ day | 830 | 117 | 107 | 117 | 250 | 1400 | 185 | 212 | 90 | 136 | 1400 | ~5000 |
| 7^th^ day | 620 | 100 | 90 | 100 | 210 | 1400 | 318 | 190 | 220 | 200 | 1600 | ~5000 |
| Concentration of proteins in dgPMCAb ^b^ | 50μM | 10μM | 2.5μM | 5μM | 4μM | 20μM | 1μM | 1μM | 1μM | 1μM | 28μM | 28μM |

^a^ ThT fluorescence is presented as counts per seconds x10^3^. Because intensity of ThT fluorescence depends on molecular structure of fibrils, ThT reading should not be used for comparing the relative amounts of fibrils present in each preparation.

^b^ Concentration of proteins added as seeds to dgPMCA reactions (presented as the final concentration in the reaction volume as calculated per monomer of a protein).
