# Supplementary figures and images for: Cross-seeding of prions by aggregated α-synuclein leads to transmissible spongiform encephalopathy
Source: PLoS Pathog. 2017 Aug 10;13(8):e1006563. doi: 10.1371/journal.ppat.1006563 (PMC5567908; doi:10.1371/journal.ppat.1006563)

Figure S1

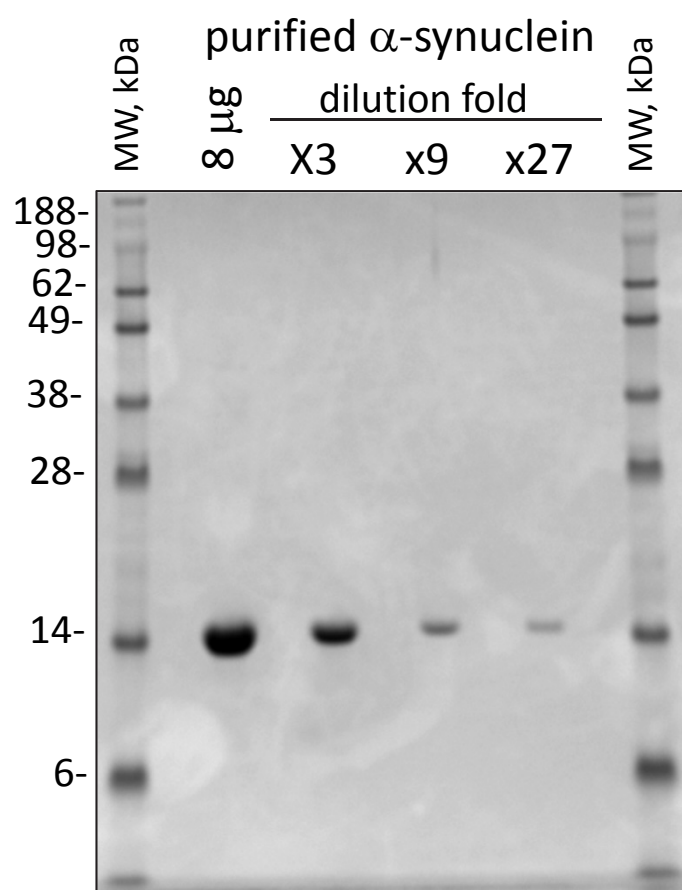

Supplement: S1 Fig — (PDF) [file ppat.1006563.s002.pdf]

Figure S2

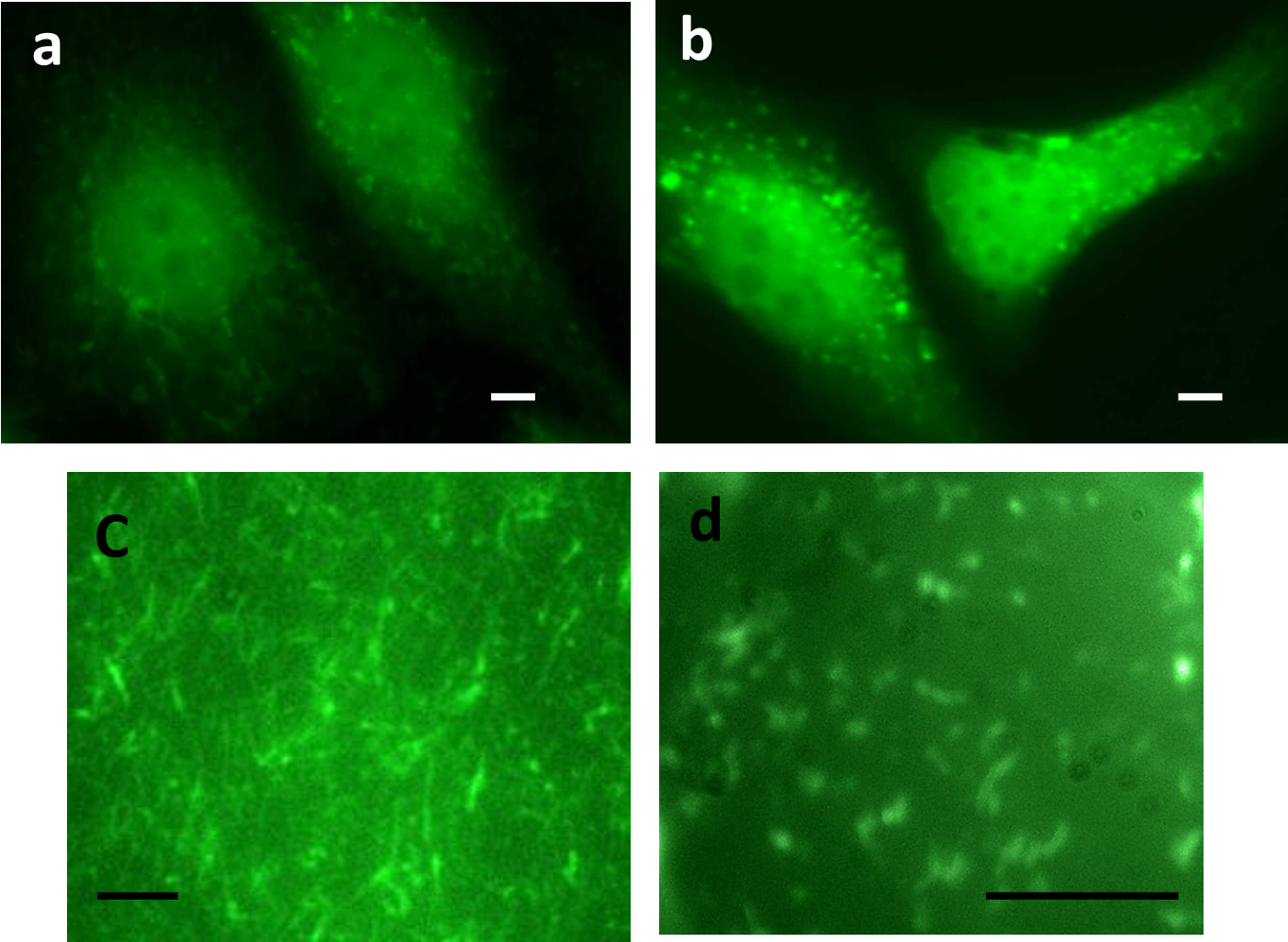

Supplement: S2 Fig — Fluorescence microscopy imaging of HeLa cells expressing human WT α-synuclein (a) or A30P variant α-synuclein (b) detected by GFP fluorescence. Fluorescence microscopy imaging of amyloid fibrils prepared in vitro using human WT α-synuclein (c) or Aβ peptide (d) and stained with Thioflavin T. Thioflavin T staining and microscopy imaging of amyloid fibrils was performed as described earlier [74]. Scale bars = 5 μm. (PDF) [file ppat.1006563.s003.pdf]

Figure S4

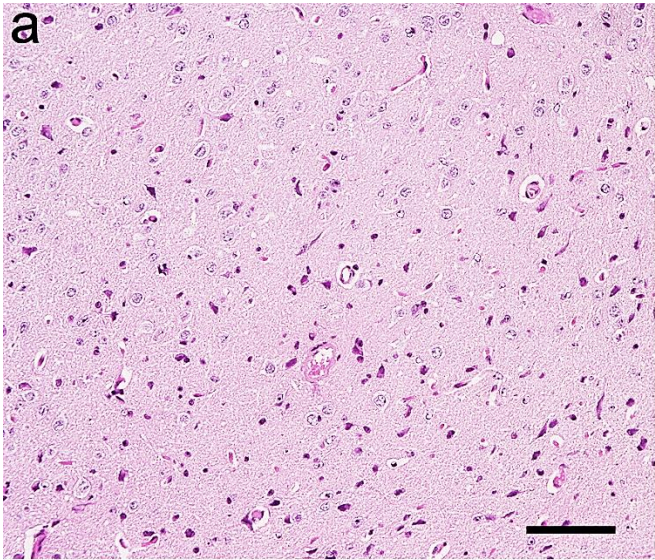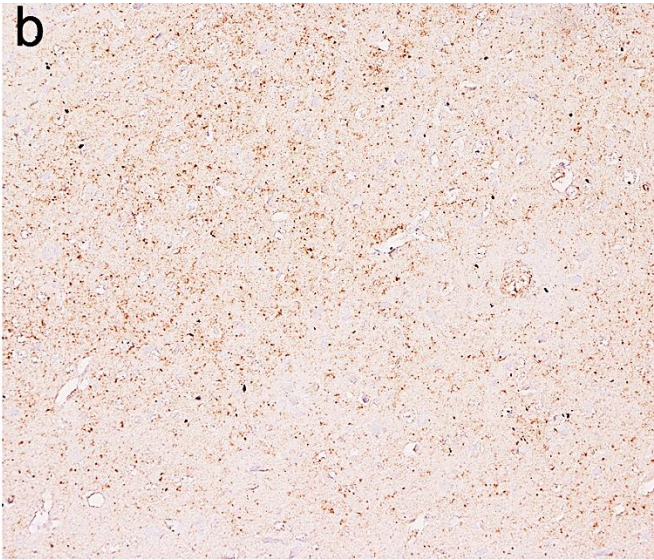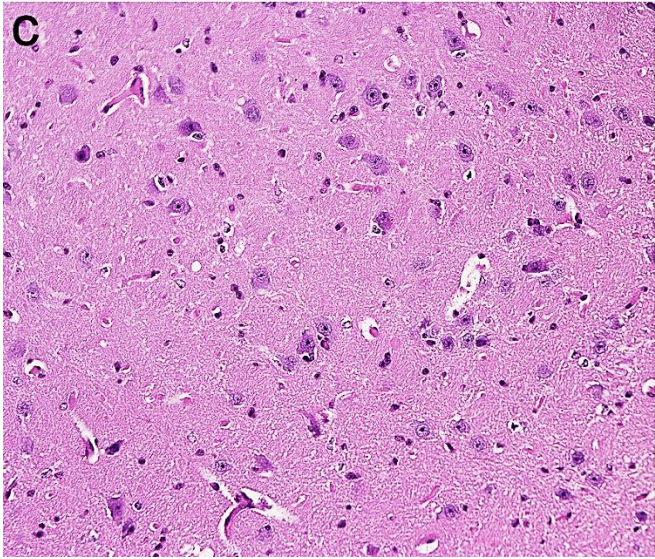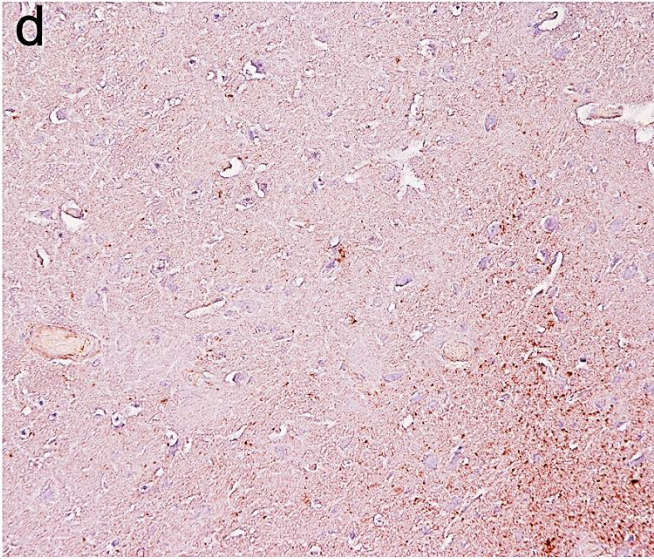

Supplement: S4 Fig — Representative images of the thalamus of animals inoculated with dgPMCAb products seeded with fibrillary α-synuclein (a, b) or dgPMCAb products seeded with lysates of HeLa cells expressing A30P α-synuclein (c, d). Note the lack of spongiform change in the sections stained with hematoxylin and eosin (a, c). Immunostaining for PrP using SAF-84 (b, d) revealed diffuse/synaptic and granular deposits. Scale bar in a = 50 μm. (PDF) [file ppat.1006563.s005.pdf]

Figure S5

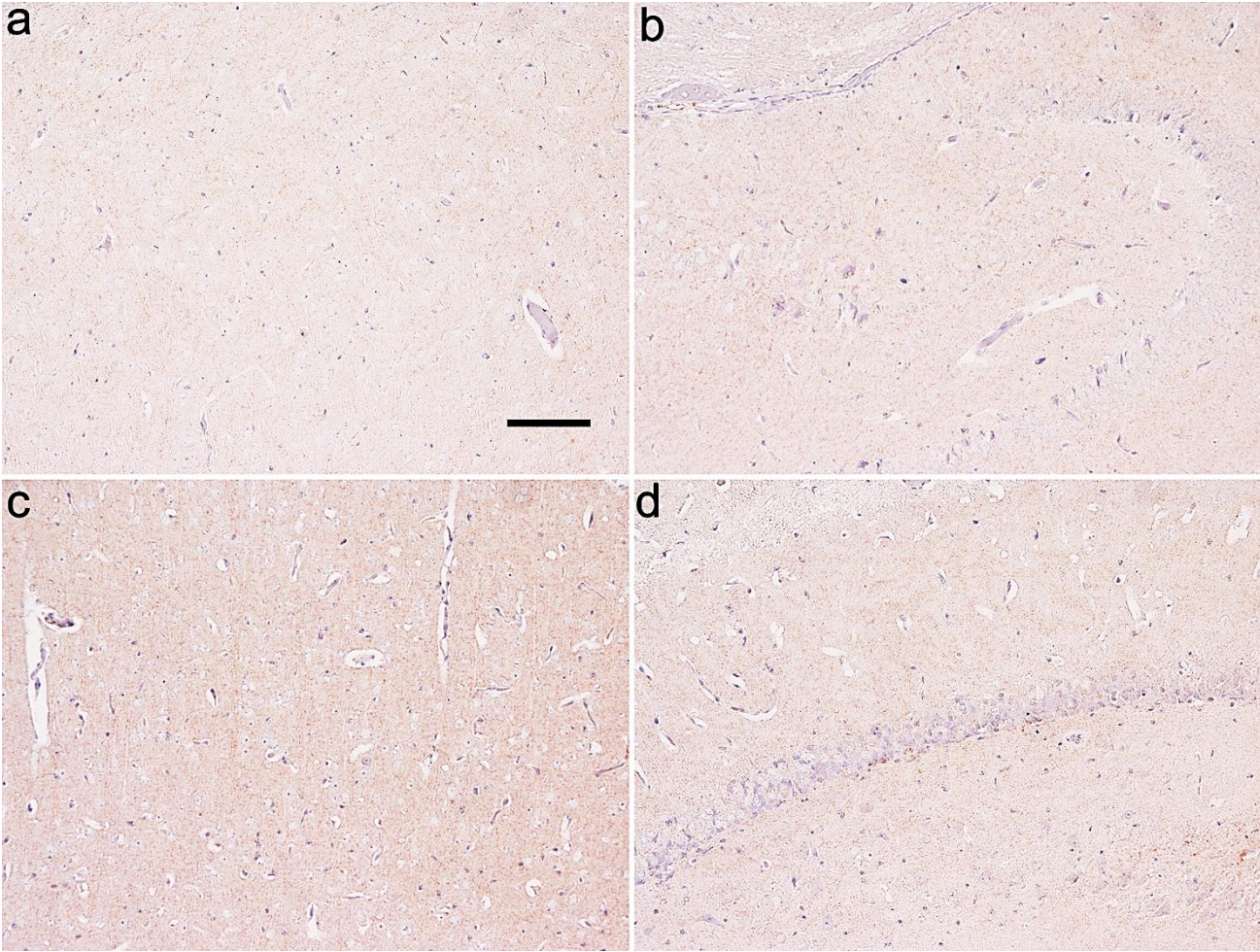

Supplement: S5 Fig — Representative images of the frontal cortex (a, c) and hippocampus (b, d) of animals inoculated with dgPMCAb products seeded with fibrillary α-synuclein (a, b) or dgPMCAb products seeded with lysates of HeLa cells expressing A30P α-synuclein (c, d) and stained with 3F4 antibody. Scale bar in a = 50 μm. (PDF) [file ppat.1006563.s006.pdf]

Figure S6

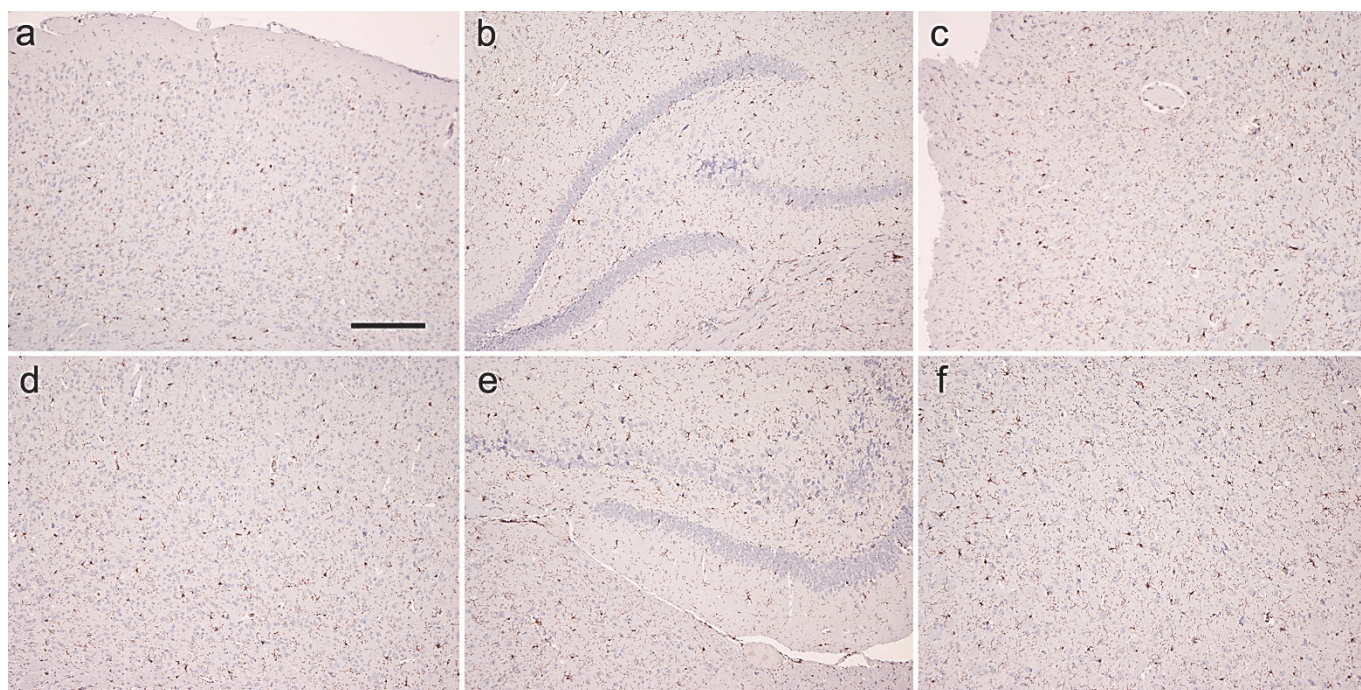

Supplement: S6 Fig — Representative images of the frontal cortex (a, d), hippocampus (b, e) and thalamus (c, f) of animals inoculated with dgPMCAb products seeded with fibrillar WT α-synuclein (a-c) or dgPMCAb products seeded with lysates of HeLa cells expressing A30P α-synuclein (d-f). Scale bar in a = 50 μm. (PDF) [file ppat.1006563.s007.pdf]

Figure S7

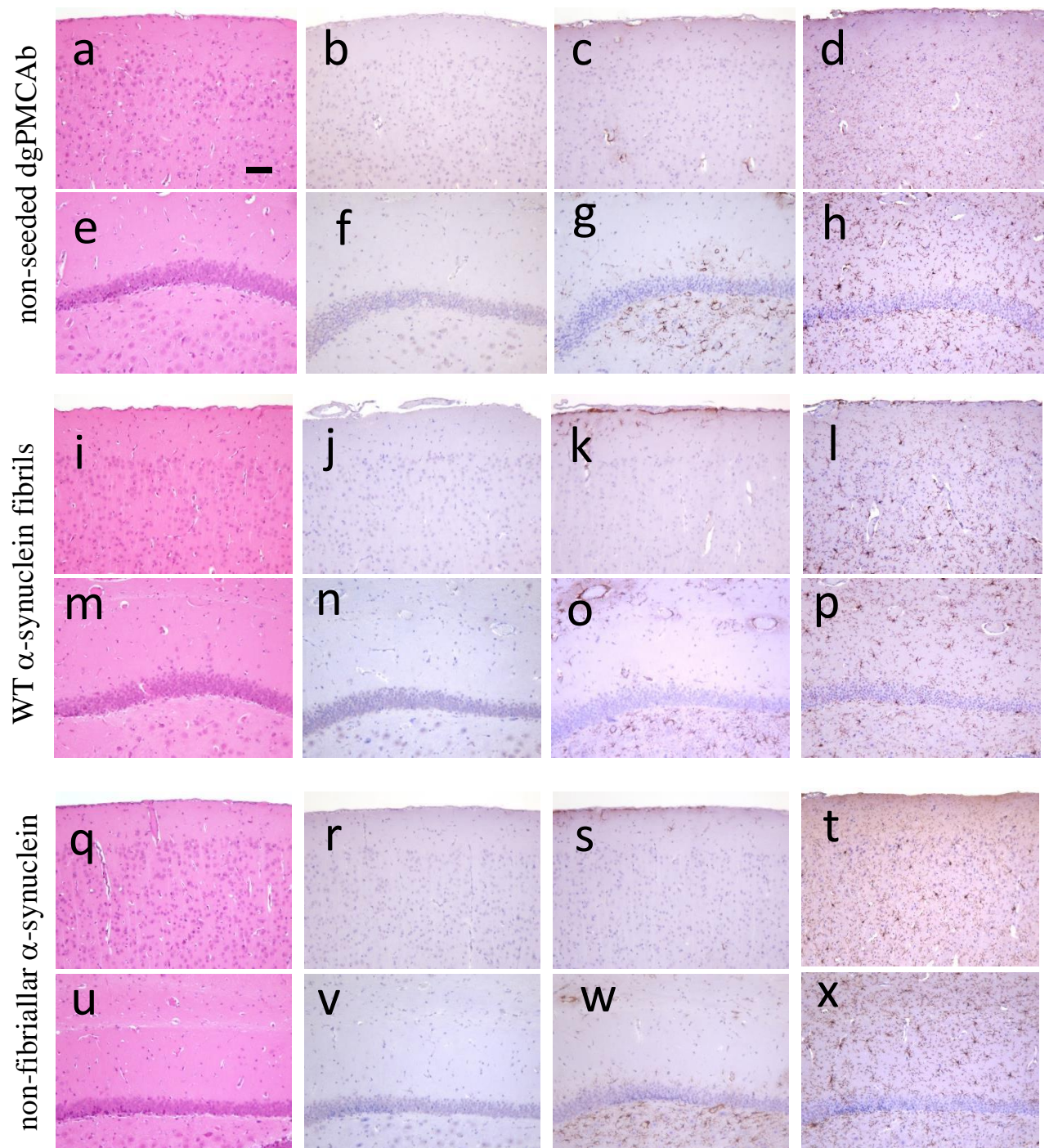

Supplement: S7 Fig — Representative images of the frontal cortex (a-d, i-l, q-t) and hippocampus (e-h, m-p, u-x) stained with hematoxylin and eosin (a, d, g, j, m, p), anti-PrP SAF-84 antibody (b, f, j, n, r, v), anti-GFAP antibody (c, g, k, o, s, w) or anti-Iba1 antibody (d, h, l, p, t, x) Scale bar = 100 μm. (PDF) [file ppat.1006563.s008.pdf]

Figure S7

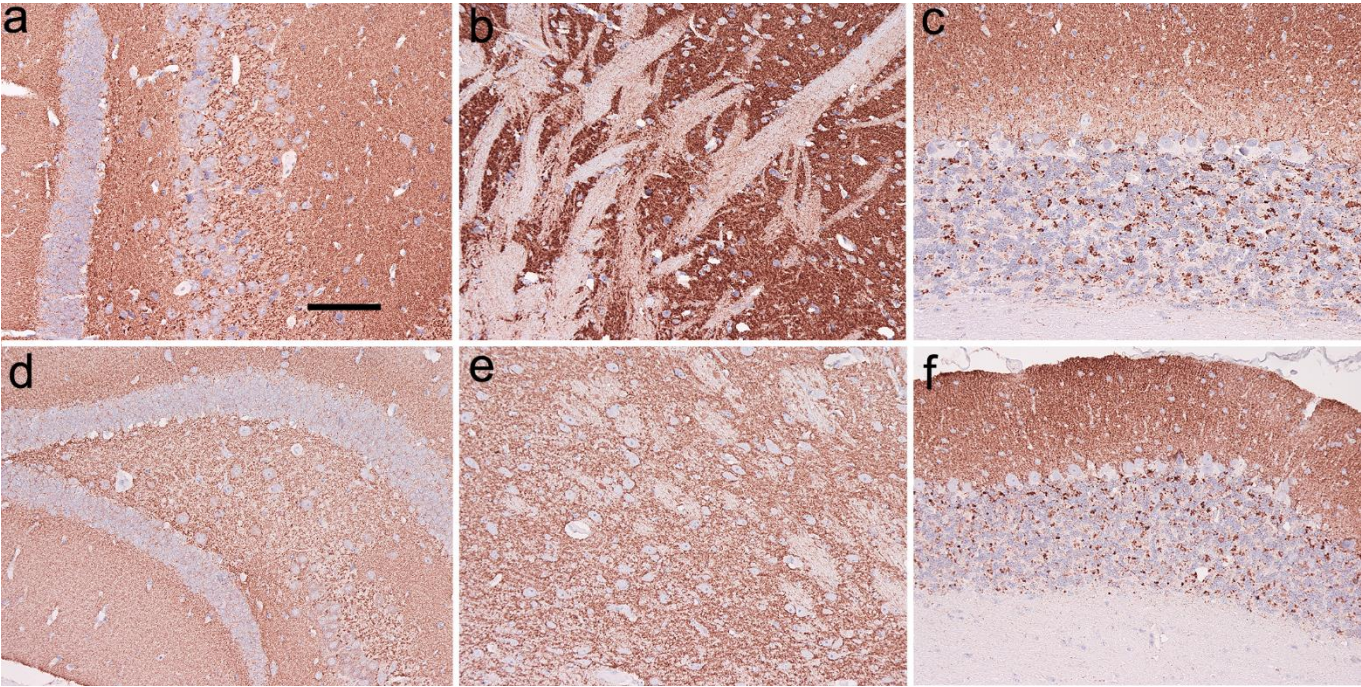

Supplement: S8 Fig — Representative images of the hippocampus (a, d), caudate-putamen (b, e) and cerebellum (c, f) showing the physiological synaptic immunostaining for α-synuclein using the 4D6 antibody. Three animals from each group were examined. Scale bar in a = 50 μm for a-c and 25 μm for d-f. (PDF) [file ppat.1006563.s009.pdf]

Figure S9

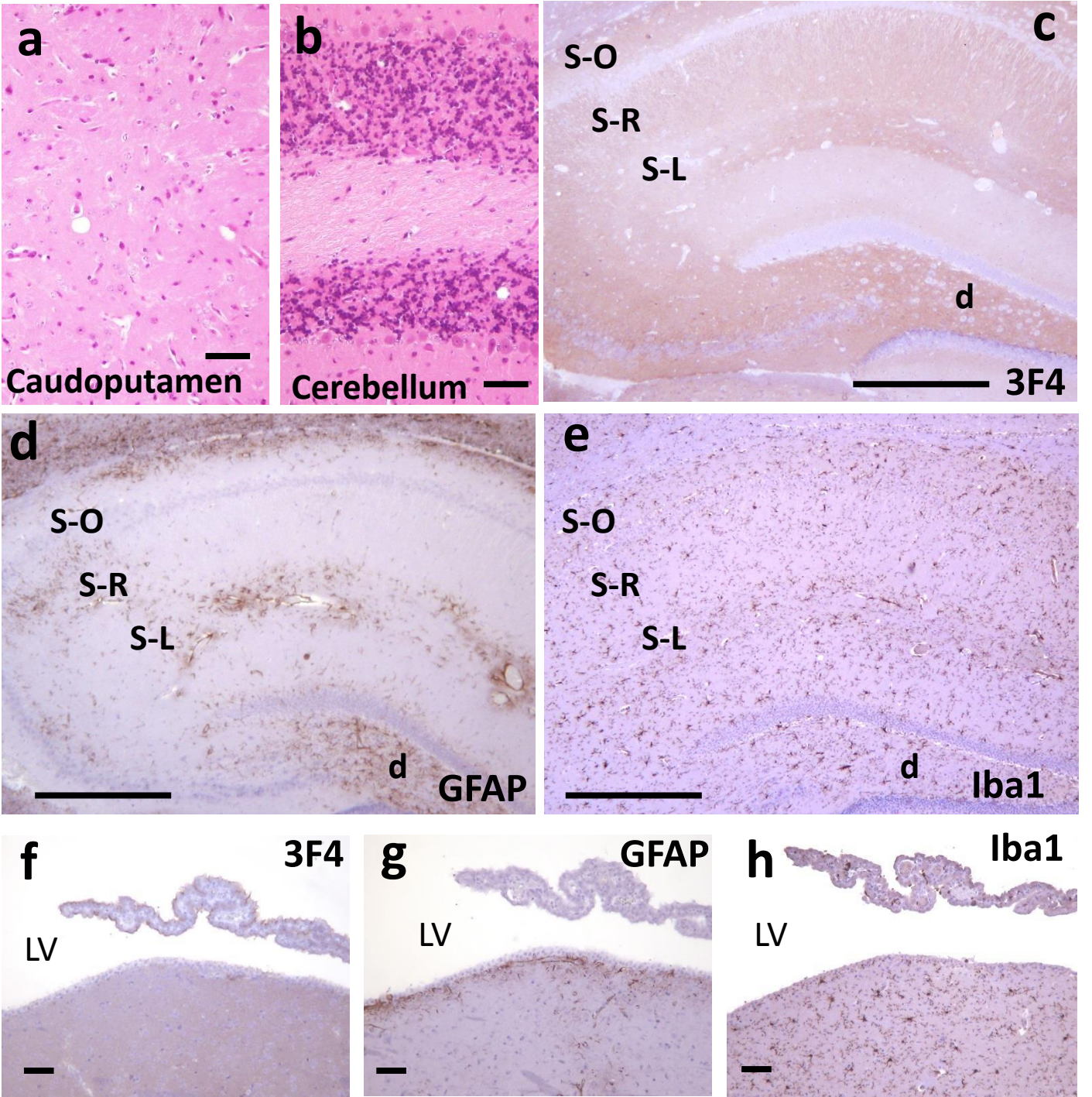

Supplement: S9 Fig — S-O, stratum orients; S-R, stratum radiatum; S-L, stratum lacunosum-moleculare; d, dentate gyrus, LV, lateral ventricle. Scale bars: in a, b, f, g, h = 100 μm, c, d, e = 500 μm. (PDF) [file ppat.1006563.s010.pdf]
