# Supplementary material for: Cross-seeding of prions by aggregated α-synuclein leads to transmissible spongiform encephalopathy
Source: PLoS Pathog. 2017 Aug 10;13(8):e1006563. doi: 10.1371/journal.ppat.1006563 (PMC5567908; doi:10.1371/journal.ppat.1006563)
Supplement: S3 Fig — (PDF) [file ppat.1006563.s004.pdf]

Figure S3

Amino acid sequences of prion proteins and α-synuclein

```
Human      23    KKRPKPGGWNTGGSRYPGQGSPGGNRYPPQGGGWGQPHGGGWGQPHGGGWGQPHGGGWG 82
Hamster    23    KKRPKPGGWNTGGSRYPGQGSPGGNRYPPQGGTWGQPHGGGWGQPHGGGWGQPHGGGWG 82
Mouse      23    KKRPKPGGWNTGGSRYPGQGSPGGNRYPPQG-GTWGQPHGGGWGQPHGGSWGQPHGGSWG 81

Human      83    QPHGGGWGQGGGTHSQWNKPSKPKTNMKHMAGAAAAGAVVGGLGGYVLGSAMSRPIIHFG 142
Hamster    83    QPHGGGWGQGGGTHNQWNKPNKPKTSMKHMAGAAAAGAVVGGLGGYMLGSAMSRPMLHFG 142
Mouse      82    QPHGGGWGQGGGTHNQWNKPSKPKTNLKHVAGAAAAGAVVGGLGGYMLGSAMSRPMIHFG 141

Human      143   SDYEDRYYRENMHRYPNQVYYRPMDEYSNQNNFVHDCVNITIKQHTVTTTTKGENFTETD 202
Hamster    143   NDWEDRYYRENMNRYPNQVYYRPVDQYNNQNNFVHDCVNITIKQHTVTTTTKGENFTETD 202
Mouse      142   NDWEDRYYRENMYRYPNQVYYRPVDQYSNQNNFVHDCVNITIKQHTVTTTTKGENFTETD 201

Human      203   VKMMERVVEQMCITQYERESQAYYKRGSS 231
Hamster    203   VKMMERVVEQMCVTQYQKESQAYYDGRRS 231
Mouse      202   VKMMERVVEQMCVTQYQKESQAYYDGRRS 230


α-synuclein  1   MDVFMKGLSKAKEGVVAAAEKTKQGVAEAAGKTKEGVLYVGSKTKEGVVHGVATVAEKTK
                 EQVTNVGGAVVTGVTAVAQKTVEGAGSIAAATGFVKKDQLGKNEEGAPQEGILEDMPVDP
                 DNEAYEMPSEEGYQDYEPEA          140
```
